# Selective Gold Recovery from Waste Electronics: A Speciation-Based Recycling Approach

**DOI:** 10.3390/ma19030538

**Published:** 2026-01-29

**Authors:** Jan Karl Ormuž, Irena Žmak, Lidija Ćurković

**Affiliations:** Department of Materials, Faculty of Mechanical Engineering and Naval Architecture, University of Zagreb, 10000 Zagreb, Croatia; jan.ormuz@fsb.unizg.hr (J.K.O.); lidija.curkovic@fsb.unizg.hr (L.Ć.)

**Keywords:** waste electrical and electronic equipment (WEEE), mechanical recycling, physical separation, hydrometallurgy, gold speciation, selective gold recovery

## Abstract

Waste electrical and electronic equipment (WEEE) is a rapidly growing waste stream rich in precious metals, with gold in particular being concentrated in printed circuit boards and other high-value components. Historically, industrial recycling has relied on pyrometallurgy and non-selective hydrometallurgical leaching. These recovery routes have major drawbacks, including high energy demand, corrosion, the use of toxic reagents, and the complexity of pregnant leach solutions, which complicate downstream gold recovery. This review aims to synthesize recent advances in selective gold recovery from WEEE using a speciation-driven approach. Mechanical pretreatment and physical beneficiation methods are critically assessed as processes for concentrating gold and reducing the amount of material sent to downstream hydrometallurgical leaching. Different lixiviants, from conventional cyanide to halide-based, as well as greener chemistries such as thiosulfate and thiourea, are assessed for gold dissolution from the WEEE stream. Assessment of different extraction methods, including sorbents, ion exchange resins, solvent/ionic liquid, direct reduction/precipitation, and electrochemical recovery, is conducted. The review concludes with guidelines for potential process integration and highlights the need for scalable, reusable lixiviants and sorbent materials validated under realistic multi-metal conditions in real WEEE leachate.

## 1. Introduction

Waste electrical and electronic equipment (WEEE or e-waste) is one of the fastest-growing waste streams globally, driven by short device lifespans and increasing consumption. According to the Global E-Waste Monitor, annual WEEE generation reached 62 million tons in 2022, nearly double the 34 Mt in 2010 [1]. This corresponds to an average growth rate of 3−5% a year, far exceeding conventional waste, global Gross Domestic Product (GDP), and population growth [2]. In terms of regions, Asia is the largest producer, reaching 24.9 Mt in 2020, according to data. It is followed by the Americas (13.1 Mt) and Europe (12 Mt), whereas Africa produces only ~2.9 Mt [3]. Despite the rising volume of WEEE, only 17.4% of WEEE is formally collected and recycled, with the majority either landfilled or processed in informal sectors [4]. The low recycling rates in some parts stem from the heterogeneity of WEEE, with many different product types, brands, and material compositions. This increases the complexity of the processes required for adequate recovery. Furthermore, there are safety concerns because of the hazardous materials that they contain, such as heavy metals, brominated flame retardants, and battery electrolytes [5]. In response, regulations such as the European Union’s WEEE Directive 2012/19/EU mandate the collection and recovery targets by WEEE category, aiming to promote the sustainable recovery of valuable materials in line with circular economic principles [6].

Among WEEE components, printed circuit boards (PCBs) and related high-grade electronic components are particularly rich in metals. PCBs constitute only 3–5 wt.% of WEEE [7], yet can contain 30–40 wt.% metals, with the remaining comprising polymers, ceramics, and glass fibers [8]. Copper is the most abundant metal in PCBs by mass. However, precious metals like gold, silver, and palladium are present at much higher relative concentrations compared to their typical levels in mining ores, specifically 1000, 250, and 110 g/ton of PCB, respectively [9,10]. According to data, one metric ton of mobile phone PCBs can contain 300–400 g of gold, vastly outpacing the 5–10 g/ton of mined ore [10,11]. Considering that in mobile phones gold constitutes less than 1 wt.% of the total mass, yet accounts for 75% of the total value, it makes a strong economic case for its recovery [12].

However, realizing this value is challenging, especially because gold in electronics is typically present at the microscale as coatings or alloyed in small components such as bond wires or thin films in transistors [13,14]. This means that bulk separation of gold is impractical unless the device is destroyed. Moreover, WEEE contains other metals that interfere with gold dissolution unless they are removed prior to said dissolution [15,16].

In industrial recycling, high-gold-bearing electronic scraps, printed circuit boards (PCBs), central processing units (CPUs), graphics processing units (GPUs), random access memory modules (RAMs), and motherboards are a preferred stream, and according to the gold content, WEEE feed is categorized into different grades: low grade (<100 ppm), medium grade, and high grade (>400 ppm) [17].

Historically, industrial recycling of e-waste has relied on pyrometallurgy and non-selective hydrometallurgical leaching akin to those in the mining sector [18]. These approaches can achieve high overall metal recovery, but they suffer from serious drawbacks.

Pyrometallurgy involves smelting WEEE feed at high temperatures, which requires substantial energy inputs and can result in the release of toxic emissions, such as dioxin and furan, if not properly controlled [19]. Also, the final product is a complex metal alloy, which requires further processing [20]. Non-selective hydrometallurgical leaching (such as aqua regia or cyanide) tends to dissolve everything, including base metals, consumes large amounts of reagents, and yields a pregnant solution filled with impurities that complicate downstream gold isolation [21,22]. In addition, cyanide is a highly toxic chemical, which poses acute environmental and health risks [23]. On the other hand, acids like aqua regia are extremely corrosive and produce hazardous waste streams [24]. These health and ecological concerns have driven research to study more selective, sustainable processes for gold recovery from WEEE, ones that operate under milder conditions, avoid the use of toxic substances, and minimize waste.

Prior reviews have evaluated the hydrometallurgical recovery of gold from e-waste [10,25] and have provided in-depth studies of different leaching chemistries [26,27] or adsorption methods [28]. Nonetheless, a clear, speciation-focused review and synthesis framework that incorporates downstream recovery within an integrated flowsheet still remains underdeveloped.

This article is a narrative-style review developed through an iterative literature search and targeted screening, with primary focus on hydrometallurgical recovery of gold from WEEE streams. Relevant literature was primarily identified using the Web of Science Core Collection database, where a broad search was conducted to identify the different hydrometallurgical routes for gold recovery. A combination of search terms, including hydrometallurg*, leaching, gold, WEEE/e-waste, and printed circuit board*/PCB* was used. As the review focus evolved, targeted follow-up searches on pretreatment and beneficiation methods for WEEE were conducted to identify studies reporting gold concentration and enrichment in pretreated waste fractions and to understand how pretreatment influences downstream recovery. Subsequently, the search for gold recovery from hydrometallurgical leachates, especially focusing on sorbent-based methods, was narrowed. In later stages, additional non-sorbent hydrometallurgical recovery methods were consulted to enable a comparative discussion against sorbent-based systems. Searches were conducted for publications from 2020 to 2026 and were primarily limited to open-access articles. A small number of relevant closed-access, peer-reviewed scientific papers were also consulted via institutional access. Records were first screened by title and abstract and then by full text. Studies were included in the review if they addressed Au dissolution from WEEE in different leachates, provided mechanistic, speciation-based insight into Au extraction from pregnant leachate solutions, and reported performance metrics such as leaching efficiency, Au uptake capacity, selectivity, or regenerability of extraction systems.

This study aims to link gold’s chemical form in different solutions with appropriate sorption or reduction mechanisms, serving as a design guide for the next generation of e-waste recycling flowsheets.

Section 2 reviews different pretreatment methods for concentrating gold from mixed streams of WEEE and for removing materials that could interfere with downstream recovery processes. In Section 3, different lixiviant systems for dissolving gold are discussed in depth, ranging from conventional cyanide-based methods to more sustainable thiourea and thiosulfate leaching. Section 4 reviews gold recovery from various leachates based on gold speciation, providing insight into recovery methods for selective gold extraction against competing metals. In Section 5, a comparative discussion is presented, in which potential recycling frameworks for gold recovery have been evaluated and proposed. Finally, Section 6 concludes with an outlook on future research and development needs to achieve greener, more economically viable gold recovery from WEEE.

## 2. Pretreatment and Beneficiation of E-Waste for Gold Recovery

**Figure 1:**
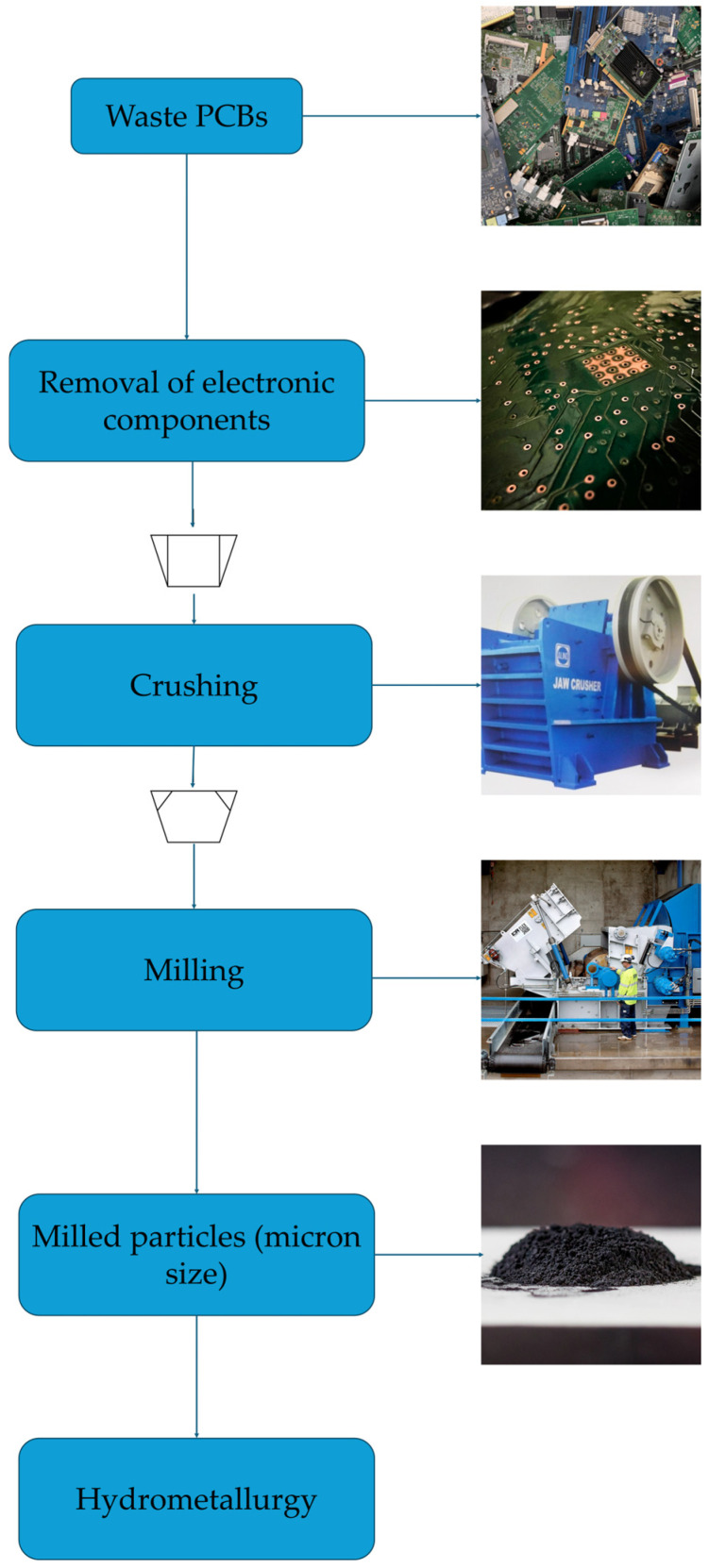
Conventional pretreatment workflow for waste printed circuit boards (PCBs) prior to hydrometallurgical gold recovery (adapted from [29] under CC BY 4.0).

Before leaching, e-waste requires pretreatment to concentrate the gold and remove or reduce the components that interfere with the downstream processing (Figure 1). Pretreatment includes mechanical, physical, thermal, and chemical processes for liberating metals from non-metallic materials such as polymers and ceramics. The final objective is to increase the gold concentration in the feed before leaching, while reducing the mass and complexity. For example, manual removal of components with low metal content, such as plastic encasings and steel screws, from high-value PCBs or integrated circuits (ICs) can increase the average gold content in the feed by an order of magnitude.

Pretreatment objectives can be grouped into (i) early separation of Au-bearing components, (ii) liberation of metals by comminution or delamination, (iii) physical upgrading by size, density, conductivity, etc. Overall, there is no universally applicable method. Rather, WEEE pretreatment is a combination of sequential pretreatment operations, dependent on downstream leach chemistry and recovery. Table 1 summarizes common pretreatment methods for WEEE, their typical operations, impact on gold distribution, and key features.

WEEE recovery begins with choosing between manual dismantling and complete comminution of electronic devices, weighing the trade-offs between the two. Manual dismantling of devices segregates valuable components, such as PCBs and RAMs, from low-value plastic enclosures and hazardous components, such as batteries. For discarded hard disk drives (HDDs), manual dismantling and separation of gold-bearing components concentrates the gold to an average of ~2187 ppm Au, whereas shredding of the whole HDDs dilutes it to ~312 ppm Au. Overall, manual disassembly leads to a sevenfold increase in the gold grade of the feed for subsequent precious metal recovery [30]. This targeted approach reduces downstream processing of low-value materials and helps minimize gold losses. On the other hand, shredding whole devices is a high-throughput method but can lead to metal losses. Therefore, modern e-waste facilities use a hybrid approach, critical disassembly, followed by mechanized crushing and separation [31]. Novel, computer-assisted methods, such as vision- or robotic-based sorting, are also increasingly discussed as a direct way to generate Au-rich streams before shredding [8].

After initial dismantling, mechanical comminution, and size reduction, e-waste is broken into particles to release embedded metals. Shredders (low-speed shear cutters) cut whole boards or devices into coarse pieces (150–250 mm) while limiting fine dust generation [48]. These pieces then pass through to crushers or mills for finer liberation. Jaw or cone crushers (compression-based) and hammer mills (impact-based) are common equipment. They operate on the principle of compression, producing relatively uniform particles with low ultra-fine dust generation, whereas high-impact milling (e.g., hammer mills) provides more complete liberation but results in more dust and fines [49]. In practice, a two-stage size reduction (coarse shred, then fine mill) is often used. While fine grinding can increase the material’s surface area and thereby enhance leaching kinetics, it is not always the best option. Comparative assessments show that aggressive comminution can increase Au losses to dust and fine-particle streams, even in the resulting metal-rich concentrates [41]. Aerosol measurements verify this, highlighting shredding and grinding stations as key sources of nanosized particle formation. [50]. As an emerging alternative to mitigate such losses, cryogenic milling (cryo-milling) processes pre-shredded WPCB particles under liquid nitrogen cooling (down to −140 °C). In a recent study on low-grade WPCBs, the approach achieved >99% mass retention and enabled systematic screening of milled particles from 4 to 0.045 mm [17].

Physical separation techniques are often used after mechanical comminution to further concentrate the gold. Screening by size is the first step. Finer fractions <0.5–1 mm were found to have higher concentrations of gold. This can be inferred from the presence of gold in smaller components, such as solder fragments and connector plating [32]. However, fine fractions also contain substantial amounts of fiber and plastic dust, which can interfere with leaching by adsorbing gold [51]. Therefore, additional separation is needed. Magnetic separation (usually by drum magnet or over-belt magnet) removes ferromagnetic iron, protecting downstream equipment, and slightly concentrating non-ferrous metals [52]. On the other hand, wet magnetic separation can also be used on fine PCB dust. The addition of surfactant octyl phenol ethoxylate to the water slurry effectively dispersed <2 mm ferrous particles, which could then be magnetically extracted, achieving 83% separation efficiency for the recovery of major economic elements [39]. Next, eddy current separators eject high-conductivity metals (Al, Cu) from non-conductive plastics, but this method is effective primarily on particles >5 mm [53]. For finer particle sizes or mixed plastics/metals feed, electrostatic separators can be used, where the particles are electrostatically charged and subsequently deflected by a strong electric field. Electrostatic methods can isolate a metal-rich concentrate (metals vs. polymers), but they are not exclusively selective for gold. Nevertheless, parameter-optimized electrostatic separation has been reported to drive most Au/Ag/Pd into conductive and mixed streams, achieving 95–96% Au recovery under optimal conditions. This created two waste streams for subsequent Au leaching [40]. Dry inertial separation lines with electrostatic separators have been assessed via LCA as a chemical-free route to produce Cu-rich concentrates that implicitly carry precious metals [38].

Density-based separation is a helpful method for concentrating precious metals. The particles are mixed in a fluid of intermediate density so that lighter polymer/ceramics float and heavier metals sink. Traditional dense media, such as halogenated organic liquids like chloroform, are hazardous. Aqueous sodium silicate (9.2 wt.% Na_2_O, 29.5 wt.% SiO_2_, and 61.3 wt.% H_2_O) and lithium metatungstate are considered less hazardous alternatives for separating PCB particles compared to chloroform, although lithium metatungstate still poses health and environmental concerns [34,37]. In one case, using a silicate solution (~2.4 g/cm^3^) increased the gold content in the sunken metal fraction by 46% [34]. Density separation of particles <2 mm in a lithium metatungstate medium of variable specific gravities (1.45, 1.68, 1.96, 2.67) showed gold concentrating in the densest fraction (specific gravity <2.67), with an enrichment ratio of 3 [37]. An indirect effect of the density separation method is the concentration of the polymer fraction with a higher energy density, which shows promise as an alternative fuel source [54]. Shaking tables and centrifugal concentrators are also used, especially for particles <0.5 mm. These can achieve high gold recoveries from ground PCB powder by exploiting the metals’ higher density relative to fiberglass and plastic [35]. For example, centrifugal separation of fine PCB dust yielded significant gold concentration with minimal losses. Centrifugal gravity and reverse flotation in tandem achieve >90% Au concentrate from WPCB fines, with explicit Au grades reported in the hundreds of g/t range [55]. Mechanistic modeling of settling/stratification on shaking tables by Nie et al. supports the idea that Au separates into the fine fraction, motivating either centrifugal gravity or flotation for <0.45 mm fractions [36].

Froth flotation uses a different mechanism based on the difference in surface hydrophobicity between polymer and resin particles in PCB grind, in comparison to metals. This makes them preferentially attach to air bubbles and float, leaving metals in the sink. Reverse flotation of PCB ultrafines has demonstrated significant upgrading of metals without the use of collectors/frothers, yielding dense metal concentrates suitable for downstream metallurgy [42]. Flotation, especially after a prior pyrolysis step to char the resin, has been shown to recover a metal concentrate with enriched gold [41]. Mechanical comminution before flotation can further enhance natural floatability and improve metal/non-metal separation, although Au is often inferred to follow the other metals [43]. For fines, hydrocyclone separation followed by dilution gravity methods has been shown to concentrate Au and Pd from PCB powders into a metal-rich underflow concentrate, reducing the mass sent to leaching [33]. Reducing the feed mass is preferred to reduce reagent demand in the leaching step.

There are also emerging pretreatment alternatives that avoid intensive comminution, such as microwave-assisted delamination. Their attractiveness stems from their ability to rapidly separate metal foils from non-metals, with minimal dust formation due to swelling and the removal of the polymeric matrix [47].

Thermal pretreatments are another method used to reduce organics and alter liberation. Microwave pyrolysis can generate a porous char, improving subsequent base metal leaching and enabling following Au leaching, though reported Au dissolution can remain incomplete depending on feed and conditions [45]. On mobile phone PCBs, microwave pyrolysis integrated with hydrometallurgy has been demonstrated with high overall Au recovery after sequential process steps, illustrating that adequate pretreatment influences downstream recovery [44]. Related hybrid chemical pretreatments include pyrolysis followed by low-temperature solid-state chlorination (e.g., NH_4_Cl at 300 °C) and water leaching, which can bring a substantial fraction of Au into solution as Au chloride for downstream recovery. The possible chlorination of metallic gold follows the reactions (Equations (1) and (2)) [56]:

NH_4_Cl (s) ⇌ HCl (g) + NH_3_ (g) (1)

4Au (s) + 4HCl (g) + O_2_ ⇌ 4AuCl (s) + 2H_2_O (2)

At high temperatures, centrifugal “supergravity” melt separation has been used as a rapid pre-concentration step to enrich precious metals into a Cu alloy and/or residue streams for subsequent hydrometallurgical processing [46].

Finally, pretreatment can be optimized against downstream electrochemical leaching. Selective disintegration into an Au-rich fine fraction has been shown to reduce the specific operating cost and increase throughput of electrochemical hydrochlorination (in situ active chlorine), illustrating how pretreatment concentration translates into reactor-scale economic gains [7].

In summary, effective pretreatment maximizes gold in the leach feed and minimizes chemical consumption. Studies have shown that prior Cu removal (by mechanical or chemical means) can dramatically improve gold leaching efficiency later. On the other hand, Cu as a catalyst in thiosulfate leaching increased the gold leaching efficiency. Thus, pretreatment, leaching, and recovery must be considered holistically to maximize the total recovery of gold from WEEE. In the following chapters, different leaching chemistries for gold dissolution will be discussed.

## 3. Gold Leaching Chemistry: Dissolution and Complexation

Effective gold leaching requires an oxidizing agent to oxidize Au^0^ to Au^+^ or Au^3+^, and a complexing ligand to stabilize the dissolved gold ion. Traditional cyanidation uses O_2_ to oxidize gold and to form the Au^+^ cation, which is then complexed by cyanide (CN^−^) to yield the stable dicyanide complex Au(CN)_2_^−^. The overall chemical reaction is as follows [57]:

4Au + 8CN^−^ + O_2_ + 2H_2_O ⇌ 4Au(CN)_2_^−^ + 4OH^−^ (3)

In WEEE recycling, a variety of lixiviants have been studied as safer or more selective alternatives to cyanide. This discussion is organized by the gold complexes formed (speciation) and the nature of the leaching system (whether acidic or alkaline, presence of catalysts, etc.). In the subsequent subsections, Au dissolution refers to the percentage of gold transferred from the solid waste fraction into leachate. Table 2 at the end of this chapter provides a comparative summary of major leaching methods.

### 3.1. Chloride-Based Leaching Systems

Chloride-based leaching systems are commonly used for gold extraction from electronic waste and are often referenced in the literature. These systems include both aggressive benchmark methods and alternative oxidative approaches to improve selectivity and operational safety.

#### 3.1.1. Aqua Regia Leaching

Aqua regia (AR) is a 3:1 mixture of concentrated HCl and HNO_3_ used as a benchmark laboratory lixiviant for complete gold dissolution. Nitric acid in AR oxidizes Au^0^ to Au^3+^, while chloride ions (from HCl) immediately complex Au^3+^ to form tetrachloroaurate AuCl_4_^−^, shifting the dissolution equilibrium forward (Equation (4)). The byproducts of this reaction are H_2_O and NO, which react with ambient oxygen to form NO_2_ (Equation (5)) [58]:

Au^0^ (s) + 4H^+^ (aq) + NO_3_^−^ (aq) + 4Cl^−^ (aq) → AuCl_4_^−^ (aq) + NO (g) + 2H_2_O (l) (4)

2NO (g) + O_2_ (g) → 2NO_2_ (g) (5)

Despite this, AR can dissolve gold powders from e-waste within a few hours at near-boiling temperatures. For example, complete gold dissolution in AR was reported at 3 h and 80 °C for a previously milled PCB metallic fraction. However, AR is highly non-selective, meaning it will also dissolve substantial amounts of Cu, Zn, Ni, Pb, Sn, etc., from the WEEE [59]. This not only consumes acid but also burdens the downstream recovery since AuCl_4_^−^ must be separated from a mixture of other metal chlorides. Optimal leaching conditions in aqua regia are met when oxidant availability and chloride activity are maintained, which is achieved by careful control of the solid-to-liquid ratio [60,61]. The corrosive nature of AR means any scale-up needs acid-resistant reactors and off-gas scrubbing to handle NO, NO_2_, and Cl_2_ fumes [62,63].

High reagent consumption and safety risks are obstacles to industrial AR leaching. Therefore, for effective, sustainable leaching, a two-stage approach is needed: before AR leaching, a pre-leaching step is performed to remove base metals.

Several studies employed HNO_3_ or H_2_SO_4_ pre-leaching to remove Cu, Zn, and other metals before gold leaching. For instance, using 2 M HCl + 2 M HNO_3_ with H_2_O_2_ oxidant dissolved most of the copper from a WEEE fraction, which was later recovered by electrowinning. The resulting product could then be leached by AR or other chlorinating agents [64]. In an even simpler method, 30 wt.% HNO_3_ alone at room temperature for 4 h dissolved copper effectively with negligible gold loss [65]. In another report, 3 M HNO_3_ at 30 °C dissolved base metals in 2 h with no Au co-dissolution [66]. Such selective base metal removal greatly reduces AR consumption and prevents base metal interference with subsequent recovery steps. Strong H_2_SO_4_/H_2_O_2_ leaching (1.8 M H_2_SO_4_ + 2 M H_2_O_2_) was used for 90% Cu removal from ground WPCBS in 1.5 h (at room temperature) while leaving gold untouched [67]. Pre-leaching in autoclave conditions can further boost base metal leaching rates [68]. An innovative pre-leach involved supercritical CO_2_ with H_2_SO_4_ to swell polymer and enhance acid penetration, followed by H_2_SO_4_/H_2_O_2_ leach. It was reported that this method can remove all base metals and concentrate gold from the solid [69].

Across these studies, it can be inferred that oxidative pre-leaching can remove >90% of Cu with <1% gold dissolution. This makes pre-leaching to remove base metals a critical first step in the overall leaching process, as it concentrates gold in solution and prevents interference from other metals during the extraction phase.

#### 3.1.2. Oxidative Chloride Leaching

Alternative chloride-based leachates are currently under investigation to diminish dependence on aqua regia. One widely studied system is acidified chloride with oxidants like NaClO_3_ (sodium chlorate) or NaOCl (bleach). For example, using HCl + NaClO_3_ can generate Cl_2_ in situ and dissolve gold as AuCl_4_^−^ without concentrated nitric acid. This reaction is presented in Equations (6) and (7) [70]:

NaClO_3_ + 6HCl → NaCl + 3H_2_O + 3Cl_2_ (6)

2Au + 3Cl_2_ + 2HCl → 2HAuCl_4_ (7)

Ling et al. showed that 1.2 M HCl + 0.5 M NaClO_3_ could achieve ~97% gold dissolution from smelter-refined WPCB feed in 1 h at 55 °C [70]. Operating at pH ~1–2 (less acidic than AR) and moderate heat made the process less harsh. Another example used HCl + NaClO (sodium chlorate(I)) under pressure. Using 4 M HCl with 0.067 M NaClO at 40 °C (0.34 MPa O_2_) gave 99% Au dissolution in 2 h after a prior epoxy removal and sulfuric acid pre-leach [71]. In these systems, chlorine gas and hypochlorous acid are active oxidizers, like NOCl/Cl_2_ for AR, but are potentially easier to handle by carefully controlling bleach addition. Bleach (NaOCl) plus NaCl in neutral or mildly acidic solution is another route. In one study, a 200 g/L NaOCl + 3 M NaCl solution concentrated Au to ~152 ppm from crushed ore, followed by solvent extraction for 99% total recovery of gold from e-waste [72]. This illustrates that bleach can efficiently leach gold, and the pregnant solution can be processed further. Even solid chloride oxidants, like KClO_3_, have been evaluated in the chloride system. A lixiviant of 0.2 M KClO_3_ + 2 M HCl, with chitosan biopolymer added, leached all the gold from WEEE at pH < 1. The mechanism involved the oxidation of Au by chlorate to Au^3+^, followed by complexation with Cl^−^ and coordination to chitosan’s functional groups, thereby improving the kinetics [73].

Overall, chloride-based lixiviants, when combined with oxidants such as sodium chlorate (NaClO_3_) or NaOCl (bleach), have demonstrated efficacy as substitutes for aqua regia in the dissolution of gold from WEEE waste. Although these systems do not achieve complete gold dissolution comparable to aqua regia leaching, they offer notable dissolution efficiencies: 97% gold dissolved in HCl + NaClO_3_ and 99% in HCl + NaClO. Consequently, they serve as a viable alternative to aqua regia leaching, operating at milder temperatures and more neutral pH levels.

### 3.2. Halide-Based Leaching Systems

Halide-based leaching systems use Cl^−^, Br^−^, or I^−^ with appropriate oxidants for gold dissolution. Chlorine-based systems, which have been reviewed before, are the most developed technology, but problems with corrosion and safety still remain. Bromine dissolves gold quickly but is expensive and toxic, while iodine systems are milder and selective but also slower and more costly. Therefore, when e-waste is leached with halide-based lixiviants, careful preparation of the e-waste feed and specialized recovery steps are required, depending on whether the lixiviant is chloride-, bromide-, or iodide-based.

#### 3.2.1. Bromide-Based Leaching Systems

Beyond chlorine, bromine-based leaching shows promise. Bromide (Br^−^) can complex Au^3+^ as AuBr_4_^−^ (Equation (8)), and bromine (Br_2_) is a strong oxidant for gold [74]:

Au + 4Br^−^ → AuBr_4_^−^ + 3e^−^ (8)

A mixture of Br_2_ and Br^–^ in acid can dissolve gold effectively. Cui et al. reported 95% Au dissolution from waste electronics using 0.77 M Br_2_ + 1.17 M NaBr + 2 M HCl in 10 h [75]. Rao et al. achieved a similar >95% Au yield in H_2_SO_4_ + NaBr (bromide) at 70 °C in just 1 h, after performing a nitric acid pre-leach of base metals [10]. The pre-leach step underlines the need to remove Cu, which would otherwise consume bromine. Bromine leaching is powerful but non-selective as it dissolves many metals. Furthermore, Br_2_ is hazardous and volatile, so it requires containment [76].

#### 3.2.2. Iodine-Based Leaching Systems

Iodine–iodide leaching is noteworthy for its potential selectivity. Iodide (I^−^) forms strong complexes with Au (notably AuI_2_^−^ or AuI_4_^−^ depending on conditions), and if an oxidant like hydrogen peroxide or iodine (I_2_ itself) is present, gold can dissolve. The gold dissolution follows the following reaction (Equations (9)–(11)) [77]:

H_2_O_2_ + 2H^+^ + 3I^−^ ⇌ 2H_2_O + I_3_^−^ (9)

I_2_ + I^−^ ⇌ I_3_^−^ (10)

Au + 4I^−^ ⇌ AuI_4_^−^ + 3e^−^ (11)

Birich et al. found that an I_2_/I^−^ leach in acidic solution (with H_2_O_2_) dissolved approximately 79% of gold from WEEE [78].

While gold dissolution efficiency in iodine-based lixiviant is at just around 80%, opposed to near quantitative dissolution reported for chloride or bromide-based lixiviant, the advantage of iodine-based leaching systems is in lower co-dissolution of base metals. This is because iodide does not form stable complexes with base metals. Therefore, iodide leaching offers a simpler process flow, as pre-leaching of base metals is unnecessary. The downside is iodine’s cost and its volatility (I_2_ can sublimate), which can limit effectiveness unless a closed system or excess iodide is used.

### 3.3. Cyanide and Cyanide-Starved Leaching Systems

Cyanide leaching is the gold standard for primary gold ores and has also been applied to high-grade electronic scrap. In a typical alkaline cyanide leach (pH ~10–11, maintained with NaOH/lime), gold oxidizes and forms the aurocyanide complex Au(CN)_2_^−^. The process is relatively slow for WEEE, as one study reports that only about 88% of Au is dissolved from pulverized PCB in 24 h with 0.135 M NaCN (~6500 ppm). The presence of base metals is detrimental, as copper and other metals consume cyanide, forming Cu(CN)_n_ and other complexes, thereby increasing cyanide consumption and slowing gold dissolution [79]. Therefore, applying direct cyanide to untreated WEEE is ineffective and non-specific. Moreover, the toxicity of cyanide is a major concern as even 100–270 ppm of free CN^−^ is lethal to humans, and typical leaching, as previously stated, can be used up to 6500 ppm [80]. Nevertheless, when base metals are removed first, cyanide can be quite effective for residual gold. Birich et al. showed that after an acid pre-leach removed most of the Cu, a subsequent cyanidation increased gold recovery from approximately 78% to 95% [78].

Collectively, these results indicate that cyanide leaching of WEEE is controlled less by Au chemistry than by base metal content, because Cu and other metals consume cyanide and decrease effective Au dissolution. The main trade-off is between achieving high Au recovery after Cu removal and addressing the significant safety, regulatory, and detoxification challenges associated with cyanide leaching systems.

To reduce environmental risk, systems with limited cyanide have been explored. These use a low cyanide concentration in combination with another ligand to dissolve gold while minimizing free cyanide. One example is glycine–cyanide leach. Glycine (an amino acid) can complex Cu(II) to form cupric glycinate, which acts as an additional oxidant, allowing synergistic leaching in alkaline solution. Oraby et al. tested a system with 30 g/L glycine and 500 ppm NaCN, which resulted in only 37.6% Au dissolution [79,81]. However, when a glycine-only pre-leach was used for Cu removal, followed by 250–300 ppm CN (with glycine still present), Au dissolution increased to approximately 89–92%. At these low CN levels, most cyanide is tied up as Cu(CN)_n_ complexes, reducing the free cyanide concentration and thereby lowering the risk of HCN gas release and acute toxicity. However, cyanide-starved systems require longer leach times or supplemental oxidant supply (e.g., dissolved O_2_ via air/oxygen sparging) to maintain dissolution kinetics.

Cyanide-starved approaches, therefore, shift the process objective from maximizing CN concentration to minimizing free CN while maintaining Au dissolution around 90% through staged pretreatment and ligand synergy. The trade-off is a better safety profile and reduced CN inventory, but it involves increased process complexity and a greater dependence on feed conditioning.

### 3.4. Thiosulfate Leaching Systems

Thiosulfate S_2_O_3_^2−^ has emerged as one of the most-researched non-cyanide gold lixiviants, especially because it forms a stable anionic complex [Au(S_2_O_3_)_2_]^3−^ with Au^+^ in mild alkaline solution. Thiosulfate has lower toxicity than cyanide, and it can selectively leach gold over many other metals when the right conditions are met [82]. However, thiosulfate leaching is chemically complex. It usually requires a copper(II)–ammonia catalyst to oxidize gold, while ammonia helps stabilize the Cu^2+^ and Au^+^ complexes. The leach pH is typically ~9–10.5. The mechanism of gold leaching in the presence of cupric ions is described in Equation (12) [83]:

Au + 5S_2_O_3_^2−^ + Cu(NH_3_)_4_^2+^ → Au(S_2_O_3_)_2_^3−^ + 4NH_3_ + Cu(S_2_O_3_)_3_^5−^ (12)

The major difficulty is that thiosulfate can undergo side reactions to form tetrathionate, effectively self-consuming in the presence of certain metals or at an unregulated pH [84].

When applied to WEEE without prior extraction of base metals, thiosulfate often yields very low gold extraction. For example, in ammoniacal thiosulfate leaching of PCB material (with Cu^2+^ catalyst, pH ~10, 25 °C), Merli et al. observed gold concentrations in solution of only a few mg/L, at large particle sizes [83]. Leaching of very fine particles (<0.25 mm) slightly increases the dissolution of gold. On the other hand, silver and palladium were leached preferentially, suggesting that the oxidizing conditions used were too mild to dissolve gold effectively. Low gold dissolution was reported for the mobile phone motherboard e-waste feed when a prior HNO_3_ pre-leach was performed. Under an optimized Na_2_S_2_O_3_–NH_3_ system (pH 10, 50 °C, 6 h), only 1.53% Au was dissolved, indicating thiosulfate leaching can be kinetically slow or incomplete for gold from e-waste if the oxidant/catalyst system is not aggressive [85].

On the other hand, if conditions are intensified, thiosulfate can achieve high gold recovery. By increasing reagent concentrations and ensuring adequate oxidant concentration, complete gold dissolution is possible. For instance, using 1.0 M (NH_4_)_2_S_2_O_3_ + 1.0 M NH_3_, with 10 mM CuSO_4_ and aeration, researchers achieved >99% Au dissolution from Cu-loaded mobile phone PCBs in 24 h. However, in the same test, significant co-dissolution of base metals occurred, complicating downstream recovery [86]. High thiosulfate concentrations also raise costs. One way to reduce thiosulfate consumption is to use it with inexpensive glycine. A glycine–thiosulfate system after acid pre-leach achieved ~93–94% Au dissolution with only 60 mM thiosulfate (much lower than the typical ~0.1–1 M) after 48 h at 40 °C, pH 9.3. Kinetic studies indicated a mixed reaction/diffusion control with a shrinking core model in such systems. This indicates that thiosulfate is effective at leaching gold, but only if base metals are previously removed and time constraints allow it [87].

### 3.5. Thiourea and Other Sulfur-Based Lixiviants

Thiourea (NH_2_CSNH_2_) is a chemical that dissolves gold by forming a cationic complex [Au(CS(NH_2_)_2_)_2_]^+^ (gold(I)-bis-thiourea). Leaching is performed in acidic conditions (often pH ~1) with the addition of a strong oxidant such as Fe^3+^. The leaching reaction is described by Equation (13) [88]:

Au + 2CS(NH_2_)_2_ + Fe^3+^ → Au(CS(NH_2_)_2_)_2_^+^ + Fe^2+^ (13)

Thiourea leaching shows rapid kinetics, often faster than cyanide leaching. However, it decomposes to form the disulfide of formamide. As previously mentioned for other leaching systems, thiourea consumption and gold leaching efficiency depend on the prior removal of base metals.

In a two-stage approach for a small-scale plant, H_2_SO_4_–H_2_O_2_ was used to pre-leach Cu/Sn, and subsequent leaching of the residue in 20 g/L thiourea + 22.5 g/L Fe_2_(SO_4_)_3_ at pH ~0.5 achieved 85–90% dissolution of Au and Ag in 1 h [67]. Similarly, Diallo et al. developed a multistage operational flow for smartphones. In the first phase, rare earths and base metals were leached in two passes [89]. Then, thiourea (42 g/L) was used with Fe^3+^ as the oxidant to achieve 98% Au and 87% Ag dissolution in 2 h. In contrast, low gold dissolution (<15%) was achieved when thiourea (25 g/L thiourea with 0.6% *w*/*v* Fe^3+^) was directly applied to mobile phone PCB powder without a prior pre-leaching step. The low performance was attributed to competition from copper, which complexes with thiourea, consuming it. In contrast, removing more than 80% of copper increased gold dissolution to 79%. Further parameter optimization, especially increasing the thiourea concentration, led to a total gold dissolution efficiency of 85% [90].

The advantages of thiourea leaching include fast kinetics at near-ambient temperatures and lower acute toxicity than traditional cyanide systems. On the other hand, thiourea is classified as a suspected carcinogen, which may complicate regulatory acceptance on an industrial scale. Nevertheless, thiourea is a lab-proven method for gold dissolution. Also, research has developed process intensification techniques to enhance gold dissolution. Ultrasound-assisted thiourea leaching on pre-removed base metal PCB samples resulted in 85.8% of Au dissolved after 2 h at 20 °C (60 g/L thiourea, 6 g/L Fe_2_(SO_4_)_3_ oxidant) [68].

**Table 2:** Comparison of leaching methods.

Lixiviant Family	Step in Au Recovery	Typical Operating Window	Au Dissolution	Key Selectivity and Practical Notes
Aqua regia/mixed HCl–HNO_3_ (3:1 conc. HCl/HNO_3_; “mild” ~2 M HCl + 2 M HNO_3_ after Cu leach; industrial PCB etching liquors) [59,60,61,64,65]	Direct, aggressive Au dissolution to chloro-complexes, benchmark in academia, sometimes preceded by HNO_3_ pre-leach	Strongly acidic, commonly ~80–90 °C, ~1–4 h; S/L roughly ~40–250 g/L	Near-quantitative Au dissolution, sequential nitric + AR reported ≥98%	Non-selective (high Cu/Sn/Pb co-dissolution). Severe corrosion and NO_x_/Cl_2_ emissions, mature chemistry, but weak sustainability case
Oxidative acid/acid–peroxide base metal leach (pre-leach); HNO_3_; H_2_SO_4_–H_2_O_2_; HCl–H_2_O_2_ [66,67,68,91]	Pre-leach of base metals (Cu/Ni/Zn/Sn) with Au retention in residue to improve downstream Au selectivity	Examples include ~2–4 M HNO_3_ around ~30 °C for ~2 h; also, H_2_SO_4_–H_2_O_2_ systems at moderate T and defined pulp densities	Au largely retained	Effective upstream “cleaning” step, oxidative/corrosive, peroxide/ozone handling adds complexity
Halide oxidizing Au leach (Cl/Br/I systems; chlorate/HCl; hypochlorite/chlorite; iodine–iodide; bromide; some pressure chlorination variants) [70,71,72,73,75]	Cyanide-free oxidative Au leaching to halide complexes, performance improves strongly after base metal removal	Typically acidic, 25 °C to ~70 °C; ~1–10 h; some pressure chlorination at higher solids	Often high (~95–100%) when well designed, bromine systems around ~95–96% in ~10 h, chloride–oxidant systems can reach ~99%	Selectivity depends on pre-leach, otherwise, Cu/Ag co-dissolution can be high. Oxidants (Br_2_, chlorate, hypochlorite) are corrosive/strong oxidizers
Cyanide-based Au leach (conventional NaCN/KCN; glycine–cyanide and cyanide-starved variants) [78,79,81]	Benchmark Au leach, low-CN + glycine, aims to reduce cyanide dose while maintaining Au kinetics	Alkaline pH ~11, room temperature, ~24 h in many WEEE tests, CN ranges from hundreds ppm (glycine-assisted) to high-CN	Condition-dependent: single-stage low-CN glycine–CN can be poor (~tens of %), while two-stage glycine + glycine–CN + pre-leach (80–95%)	Highest industrial maturity for ores, glycine helps moderate Cu but does not eliminate base metal issues
Acidic thiourea-based Au leach (TU + Fe^3+^ in H_2_SO_4_; ultrasound-assisted variants; commonly after oxidative pre-leach) [67,85,89,90]	Non-cyanide Au/Ag leach, usually Stage 2 after base metal removal	pH ~1, ~20–60 g/L TU with Fe^3+^ oxidant, ~1–6 h; often near room temperature	~70 to >95% depending on feed/pre-leach, some smartphone/WPCB sequences show ~96–98%	Faster initial kinetics than many alternatives, thiourea degradation
Thiosulfate-based Au leach ((NH_4_)_2_S_2_O_3_ + NH_3_/NH_4_^+^ + Cu^2+^; glycine–thiosulfate synergies) [83,87,92,93]	cyanide alternative; sensitive to Cu/ammonia control; benefits from strong upstream base metal removal	pH 8–10, 25–50 °C, ~2–24 h; thiosulfate ~0.1–1.0 M with Cu catalyst in compiled cases	Highly variable, low for direct leaching, can reach >97–99% after optimization	Lower toxicity than CN, high thiosulfate consumption, complex Au recovery

**Figure 2:**
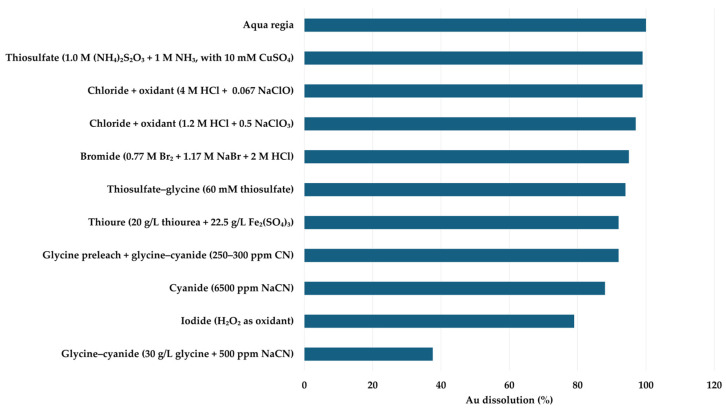
Representative gold dissolution efficiencies reported for major WEEE leaching systems (conditions and literature sources summarized in Table 2).

Figure 2 compares representative Au dissolution efficiencies for the main leaching systems discussed in this section. Different leaching systems vary in their ability to complex gold and create gold complexes suitable for the next stage of the overall gold recycling process, the gold recovery phase. This is where the speciation of gold in leachates becomes critical, as it determines the potential extraction process. In the next chapter, different extraction methods will be discussed based on leaching type and gold capture mechanisms.

## 4. Recovery of Gold from Leach Solutions: A Speciation-Driven Design

After leaching, the selective recovery of gold from pregnant solutions becomes the key factor in determining process performance and sustainability. Recovery methods must be tailored to the chemical form of gold in solution and the complexity of co-dissolved metals. As a result, many modern approaches rely on controlling gold speciation to enable selective separation or reduction.

### 4.1. Acidic Halide Leachates

Acidic halide leachates are among the most common media for dissolving gold from electronic waste, where gold mainly exists as anionic chloro-complexes. The stability of these species allows for selective recovery strategies, but the highly acidic, chloride-rich environment also increases competition with base metals and limits material stability. Various recovery methods have been developed, ranging from sorption-based capture to redox-assisted immobilization and direct reduction.

Table 3 summarizes representative sorbent materials reported in the literature for selective gold extraction from chloride solutions, comparing their feed matrices, operating conditions, adsorption performance, selectivity toward gold over competing metals, and proposed adsorption/regeneration mechanisms.

#### 4.1.1. Carbon-Based Sorbents and Graphene Nanostructures

In acidic leachates where gold is present as an anionic complex (AuCl_4_^−^), functionalized activated carbons can increase selectivity against the competing ions and enable integrated electrorecovery. An imidazolium ionic-liquid (IL)-grafted biomass carbon (ACIL900) showed a maximum adsorption capacity (*q*_max_) of about 116.2 mg/g for Au^3+^ at 25 °C. The removal of Au from real leachates was reported to exceed 99%, with preferential adsorption of Au^3+^ over Cu^2+^ following a chemisorption mechanism. The loaded sorbent was then used as a cathode for the electrochemical reduction in Au to the metallic state [94].

On the other hand, graphene-based nanostructures combine high accessibility with redox-active capture for direct in situ reduction of AuCl_4_^−^ to Au^0^.

**Figure 3:**
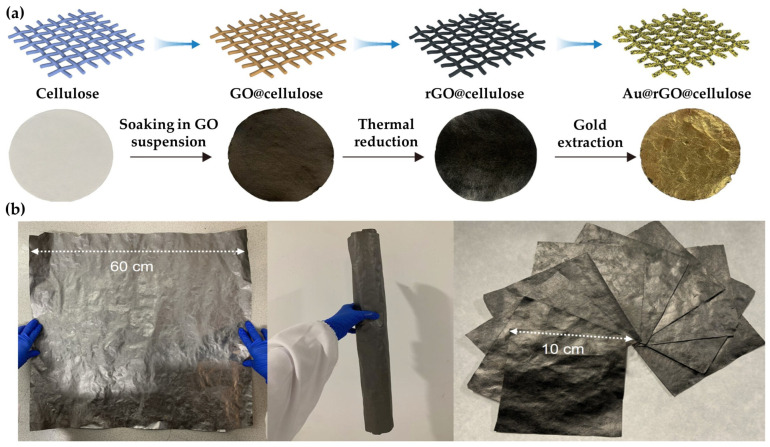
(**a**) Schematic illustration of the fabrication process of rGO@cellulose and its gold extraction process, where the lower panel shows the corresponding pictures of the materials used in each step; (**b**) photographs of large-area rGO@cellulose (adapted from [95] under CC BY 4.0).

Figure 3 shows the fabrication concept and the appearance of the graphene oxide (rGO)@cellulose composite paper before and after Au extraction. This sorbent was used as a flow-through filter with an extraction efficiency of approximately 99.6% for Au from real aqua regia PCB leachate. Across 13 monitored metals, no co-adsorption was observed, indicating high selectivity. High overall Au loading of 4660 mg/g was achieved. The proposed mechanism involved reductive deposition, in which rGO serves as an electron reservoir while Au forms a dense coating [95].

Strong selectivity for Au over Cu and Fe was observed in rGO membranes within AuCl_4_^−^ mixed-ion solutions, driven by interfacial adsorption and reduction. At pH 4, in PCB-simulated mixed-ion solution, the rGO membrane exhibited a Au uptake of 262 mg/g after 48 h, which was lower than that of rGO@cellulose [96].

The uptake of rGO can be increased by introducing sulfur- and nitrogen-bearing ligands into the nanostructure. Thiourea-functionalized rGO in model chloride solutions exhibited 833 mg/g of Au uptake and demonstrated high selectivity for Au in mixed metal solutions of Cu, Zn, Pb, and Au at pH 2 with various initial Au concentrations (10, 1, 0.1, 0.01 mg/L). This suggests that adding suitable functional groups can improve gold adsorption on rGO even at lower pH levels [97].

An alginate-derived pyrocarbon is a “green” carbon-based sorbent capable of selectively adsorbing and reducing gold across a wide pH range (pH 1–8). Reported Au uptake was 2829.7 mg/g in the temperature range 25–60 °C. In a real diluted aqua regia leachate of CPUs, 100% Au removal was achieved in 300 min. Strong chemisorption was indicated as a driving mechanism coupled with reduction by electrons donated from sp^2^-hybridized carbon sites [98].

Overall, novel carbon-based sorbents have proven effective at near-total or total Au recovery from chloride-based pregnant leachates. Reported Au extraction efficiencies include >99% (ACIL900 in real leachate followed by electroreduction to Au^0^), 99.6% for rGO@cellulose from real aqua regia PCB leachate, and 100% (alginate-derived pyrocarbon from diluted CPU aqua regia leachate).

#### 4.1.2. Biosorbents

Biosorbents are derived from renewable biological materials and are attractive for gold recovery due to their low cost, abundance, biodegradability, and rich surface chemistry. As a representative baseline system, an unmodified lignocellulosic sorbent based on cross-linked orange peel was investigated. Its maximum capacity for Au was 72.65 mg/g from single-metal AuCl_4_^−^ solutions (pH 2, 25 °C, 30 min). In mixed-metal solutions of the pyrolyzed PCB feed, a preference for Au over Cu was observed. The driving sorption mechanism is ion exchange via carboxyl/hydroxyl groups, which has been shown to be effective for stage metal separation [99].

Functionalizing biomass with thiol groups provides greater selectivity in more aggressive chloride solutions compared to unmodified lignocellulosic sorbents. In diluted aqua regia (1.3 M) at room temperature, thiol-modified garlic peel was reported to have a *q*_max_ of 42.6 mg/g for gold. It recovered more than 92% of Au within 60 min at room temperature, with Cu, Ni, and Zn below detection levels, using a sorbent dosage of 10 g/L [100].

Taken together, both unmodified lignocellulosic and thiol-functionalized garlic peel demonstrate that low-cost biomass can selectively capture gold directly from complex chloride leachates, but the adsorption mechanism is important. Oxygenated ligands facilitate the separation of stage metals, while sulfur functionalization selectively captures gold. Despite its ability to selectively target gold, the practical use of this sorbent type is limited by its low capacity and restricted long-term reusability over multiple adsorption and desorption cycles.

Shifting from minimally processed biomass sorbents to polyphenol- and tannin-rich materials shifts the focus from primarily ion exchange and complexation to a combined ion exchange and chelation approach. The tannin/polyphenol system, a phenolic polymer derived from pomegranate peel, achieved a maximum capacity of about 315.45 mg/g and removed approximately 97% of gold (Au) from a multi-metal WPCB leach simulant at pH 2. A strong preference for Au over base metals and a capacity retention of about 90% over three adsorption and desorption cycles were observed [101].

A related tannin-based chelating sorbent, the tannin–ethylenediamine derivative, reached equilibrium in 26 min and achieved a removal efficiency of 99% for both Au and Pd at approximately pH 2. It had Langmuir *q*_max_ values of approximately 220.3 mg/g for Au and 261.2 mg/g for Pd, with significantly higher distribution coefficients for Au/Pd (around 10,000 to 100,000 mL/g) compared to common base metals [102].

**Figure 4:**
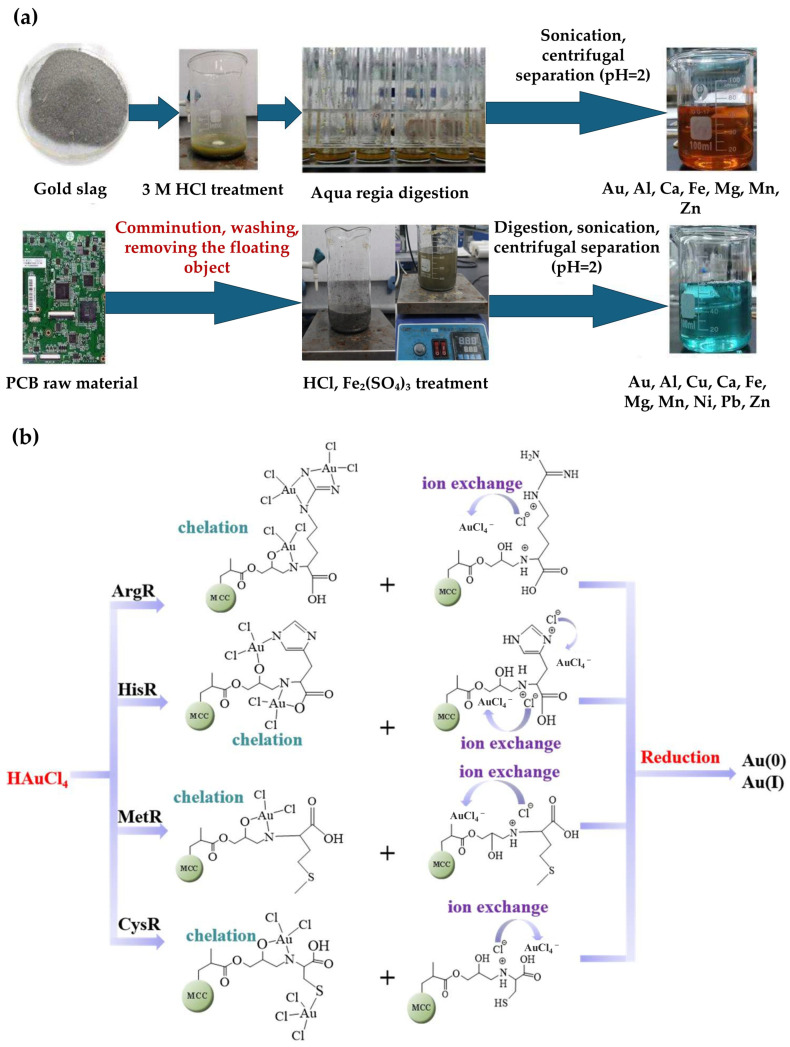
Gold adsorption on amino acid-functionalized cellulose microspheres: (**a**) gold leaching; (**b**) mechanism of Au(III) ion adsorption onto amino-acid resin (adapted from [103] under CC BY 4.0).

Amino-acid-functionalized cellulose microspheres broaden the ligand set to N/S donors and report high intrinsic capacities, with Langmuir *q*_max_ values ranging from 397 mg/g to 769 mg/g. When applied to real PCB and gold-slag leachates (Figure 4), Au removal ranged from 68.1% to 82.1%, depending on chelation and ion exchange adsorption mechanisms [103].

For a more acidic chloride-leaching system, dithiocarbonate-modified cellulose (DMC) and an epoxy-crosslinked derivative (DMC-Pro-Epo6) maintained more than 99% Au removal from up to 10 times diluted aqua regia across a wide Au^3+^ range (100 to 7000 μmol/L) at 25 °C, with minor adsorption of Cu, Ni, and Fe. At the same time, Pt and Pd were also captured, and Ag was weakly absorbed. Metallic gold is ultimately extracted through thermal decomposition of the sorbent [104].

These cellulose-based sorbents demonstrate that functionalized biopolymers can enhance capacity and preserve Au selectivity under realistic leaching conditions by combining strong soft ligand coordination, especially sulfur-containing functional groups, with improved material stability. Compared to raw biomass, the main trade-off is increased synthesis and processing complexity, which must be justified by performance under aggressive leach conditions and by simpler downstream metal recovery, such as through thermal conversion to metallic gold in the DMC systems.

At the same time, several studies demonstrate that very high capacities and fast kinetics can also emerge from upgraded waste-derived sorbents and protein-based nanostructures, provided selectivity holds in multi-metal feeds. Acid-treated kiwi peel represents a low-cost biomass with unusually high reported Au loading in acidic chloride lixiviant. In 0.1 M HCl at 30 °C, modified kiwi peel reached a *q*_max_ of about 5.71 mmol/g (about 1125 mg/g), achieving about 99% Au removal in single-metal systems and about 92% Au removal in mixed Au/Pd/Pt solutions. Gold desorption was also demonstrated in a thiourea–HCl solution with 75% efficiency [105].

Another biological waste stream was identified for gold extraction from chloride solutions. Modification of fly ash with an amyloid coating increased its capacity from about 18.9 mg/g (raw fly ash) to 58.7 mg/g at 25 °C and 400 ppm Au. Increasing the adsorption temperature to 60 °C improved adsorption, with capacity increasing to 247.5 mg/g. Furthermore, the sorbent was shown to be regenerable after subsequent thiourea–HCl desorption of Au [106].

An increase in Au concentration in chloride solutions increases the total uptake capacity, as shown for protein amyloid fibril aerogels. While uptake capacity at 1 ppm Au was just 0.12 mg/g, increasing it to 100,000 ppm resulted in a capacity of 1887 mg/g. Selective adsorption of gold was observed in a mixed-metal solution containing Cu, Ni, Pb, Zn, Cr, Fe, and Mn at 93.3%, with a gold adsorption capacity of 166.7 mg/g. Also, the aerogel exhibits fast kinetics, achieving 77% gold recovery in just 5 min. The real aqua regia motherboard leachates further confirmed the selectivity over the base metals [107].

Consistently high Au extraction efficiencies from chloride-based pregnant leach solutions were indicated from the reviewed studies. The thiol-modified garlic peel biosorbent was shown to be effective at extracting more than 92% of Au from diluted aqua regia (1.3 M) within 1 h, with negligible co-uptake of Cu, Ni, and Zn. On the other hand, a high extraction efficiency of 97% was reported for pomegranate peel-derived polyphenol sorbent from simulated multi-metal WPCV leach solution (pH 2). The sorbent demonstrated reusability, with a retained capacity of about 90% across three adsorption/desorption cycles. Near-quantitative Au removal efficiency was achieved using a tannin-ethylenediamine derivative from chloride solutions at pH 2. Amino-acid-functionalized cellulose microstructures achieved 68.1–82.1% Au removal from real PCB and gold-slag pregnant leach solutions, while dithiocarbonate-modified cellulose systems maintained over 99% Au removal from tenfold diluted aqua regia across wide initial Au concentrations, enabling downstream recovery of metallic gold through thermal decomposition. Waste-derived and protein-based sorbents also show high Au removal efficiency. Acid-treated kiwi peel was effective at 99% Au removal in single-metal solutions and 92% Au removal in mixed-metal solutions (Au/Pd/Pt). Furthermore, around 75% desorption efficiency was reported with thiourea–HCl solution. Selective Au removal at 93.3% efficiency was observed from a mixed base metal solution using amyloid fibril aerogels [100,101,102,104,105,107].

#### 4.1.3. Covalent Organic Frameworks and Related Covalent Organic Sorbents

Visible-light-active COF isomers demonstrate that framework stacking can directly affect both capacity and AuCl_4_^−^ capture. Benzothiadiazole-based Tp-BTD COF, where Tp denotes 1,3,5-triformylphloroglucinol, and BTD denotes benzothiadiazole, the AA-stacked (eclipsed) isomer delivered the highest uptake capacity (about 3094.6 mg/g under 460 nm light compared to 1706.5 mg/g in the dark). The AB and ABC stacking arrangements (lateral shifts) resulted in reduced capacity, consistent with lower accessible surface area in the more constrained stackings. Selectivity remained strong in mixed-ion solutions, and AA removed 97.8% Au from a near-neutral CPU leachate (3.67 ppm Au, 732.8 ppm Cu, 50.4 ppm Ni) with little Cu/Ni uptake. Sorption follows a photo-redox chemisorption mechanism where the COF reduces Au^3+^ mainly to Au^0^ on adsorption sites [108].

Across these COF isomers, light was identified as an influencing factor. At the same time, stacking-controlled accessibility explains that a COF built from a single building block can exhibit performance differences. Furthermore, while reported capacities were impressive, scaling up photochemical sorbents requires control of irradiation and consideration of mass transfer.

Shifting the leaching conditions from mild to acidic (aqua regia) requires a shift from photoactivation to heteroatom-rich frameworks with fast chemisorption and reduction sorption mechanisms.

Benzoxazine-based COFs showed high Au capacities (maximum capacity of 3467 mg/g) in aqueous AuCl_3_ solutions with varied initial concentrations from 1 to 2000 mg/L. Adsorption performance was nearly 100% within the acidic pH range (2–6). The equilibrium was reached in 10–30 min, showing fast kinetics in a 100 mg/L HAuCl_4_ solution. Across multiple adsorption/desorption cycles for different variants of the framework, no notable performance loss was noted. In real PCB leachate, Au recovery exceeded 92% with minimal co-adsorption of Cu, Fe, Ni, Zn, and Al, and selectivity remained high even when base metal concentrations were increased by 100 times [109].

Another approach is to functionalize an ionic COF with groups that enable ion exchange and use it as a membrane. TpTGCl-based (where TpTGCl refers to a triformylphloroglucinol–triaminoguanidinium chloride framework) cellulose–COF nanopapers maintained a capture capacity of about 1794 mg/g and achieved high selectivity in a flow reactor (in a 50 ppm Au plus 50 ppm Cu feed, Au removal was 99.9% while Cu removal was only about 2–4%). In a flow-through reaction, 99% Au removal was noted for the first 200 mL, while total removal for 505 mL was 74% to 87%, dependent on the flow rate of the mixture. In real PCB-aqua regia leachate, Au removal exceeded 90% with no co-adsorption of other metals, showing high selectivity towards Au. The reported mechanism is chloride counter-ion exchange for AuCl_4_^−^, followed by reduction to Au^0^ within the COF [110].

Under acidic chloride conditions, COF performance is governed by (i) strong anion-focused capture (chelation or ion exchange) and (ii) reduction to metallic Au^0^, which suppresses competing ions.

Related covalent organic polymers and microporous redox polymers exhibit the same adsorption and reduction principle without the crystalline COF structure. A fluorinated imine-based covalent organic polymer (N-TFACOP) has a very high capacity with broad selectivity. Capacity reached about 2975 mg/g at pH 4 and 45 °C (720 min) and about 2037 mg/g at pH 6 (25 °C), with more than 99.9% removal from 10 ppm Au^3+^ within 24 h at pH 4 and negligible uptake of competing cations. Desorption using thiourea with HCl maintained about 98.9% recovery over six cycles [111].

Across COFs and closely related covalent organic sorbents, high Au selectivity in chloride leachates is repeatedly driven by coupled anion-selective capture and in situ reduction of AuCl_4_^−^ to Au^0^. The key parameters influencing gold adsorption were stacking/channel transport and the redox mechanism (photo-enhanced vs. intrinsic). The critical question is whether the high capacity and reusability of these sorbents can be sustained in scaled-up systems under more realistic conditions.

In summary, COFs and covalent organic polymer nanostructures typically achieve more than 90% Au extraction efficiency from chloride-based leachates. Reported examples include 98.8% Au removal from near-neutral CPU leachate using an AA-stacked photoactivated Tp-BTD COF, over 92% Au extraction from real PCB leachates with benzoxazine-based COFs and minimal co-adsorption of base metals, and more than 90% Au removal from real PCB aqua regia leachate with TpTGCl-based cellulose-COF nanopapers. Imine-based covalent organic polymer also achieves near-quantitative Au removal while showing regenerability through thiourea–HCl desorption over multiple cycles [108,109,110,111].

#### 4.1.4. Metal–Organic Frameworks

Metal–organic frameworks capture gold mainly through three mechanisms: (i) light-induced redox processes, (ii) sulfur or N/S ligand chemisorption, and (iii) fast adsorption of trace Au or Au nanoparticles. These methods have been effectively used in flow-through reactors. Visible-light-active Zr-MOFs can combine adsorption with in situ reduction to accelerate Au capture. An amino-functionalized UiO-66 MOF (NH_2_-UiO-66), asymmetrically modulated with benzoic acid during synthesis, achieved a maximum capacity of ~1040 mg/g in the dark and ~2040 mg/g under visible light within ~25 min at a wide pH range (2 to 10) while maintaining high selectivity for gold in multi-metal solutions [112].

A cyclic trinuclear unit Cu^+^ metal–organic framework (JNM-103) enhanced both kinetics and selectivity. Under visible light, it removed >99% of Au from solutions containing 500 ppm Au in approximately 20 s. In real CPU aqua regia leachates, highly selective adsorption (98%) of gold compared to Cu and Ni was reported [113].

Light-assisted MOFs’ performance mainly depends on effective electron transfer to Au species, leading to faster kinetics and higher capacities in complex feed. The trade-off is that reported yields rely on continuous light supply, which requires additional light-generating equipment for potential scale-up systems. When the leach medium is highly chloride-rich and often strongly acidic, the primary design approach shifts from photactivation to sulfur- or N/S-rich binding sites that stabilize Au capture and promote reduction even in the absence of light. Several Zr-MOFs functionalized with thiourea- or thiol-bearing ligands demonstrate high selectivity for Au in chloride media. UiO-66-AT (amidinothiourea linker) achieved a maximum experimental uptake of approximately 903 mg/g at an initial Au concentration of 1000 mg/L, with an optimal pH around 3 and reaching equilibrium in about 120 min, along with strong selectivity over common base metals and good regenerability over multiple cycles [114].

For mercaptosuccinate grafting, UiO-66-MSA reached a maximum capacity of approximately 741.8 mg/g (Langmuir *q*_max_ around 720 mg/g at 25 °C). It achieved 99–100% gold removal from solutions under optimized conditions, with strong selectivity. Also, it was reported that UiO-66-MSA maintained a capacity above 90% during repeated adsorption and desorption cycles [115]. High selectivity was also reported for thiotic-acid-functionalized UiO-66 [116]. This is attributed to a coupled sulfur-redox mechanism, where S(–II)/disulfide pairs reduce Au while forming Au–S bonds. This was demonstrated on Zr-MOF prepared with mercaptosuccinic acid and applied to real CPU leachates in 10 adsorption/desorption cycles [117].

For industrial applications, besides kinetics, selectivity, and capacity, the extraction of sorbents from solutions is also an important factor to consider. A novel magnetic nanobiocomposite based on the sulfur-functionalized Zr-based metal–organic framework was introduced. The MOF was synthesized on Fe_3_O_4_ nanoparticles, enabling magnetic removal of the particles [118].

A mesoporous mercaptobenzoinic-acid-functionalized Z-based MOF with rapid uptake kinetics was examined in near-neutral solutions (pH 6). In low ppm solutions, a *q*_max_ of 714.3 mg/g was reached for Au removal in low ppm Au solutions within about 1 min. This aligns with the role of sulfur-redox sites and open channels in enabling quick, effective Au uptake [119].

Light-assisted redox capture and sulfur/N-S chemisorption, combined with in situ reduction, enable MOF-based sorbents to achieve near-quantitative Au removal from chloride leachates. Light-activated JNM-103 MOF was effectively used in real CPU aqua regia leachates for selective Au removal (98%). Under highly acidic conditions, zirconium-based MOFs functionalized with sulfur-containing functional groups show 99–100% Au removal with UiO-66-MSA sorbent retaining more than 90% capacity over repeated adsorption/desorption cycles. Similar high selectivity and regenerability were observed for other sulfur-functionalized UiO-66-based sorbents in real CPU leachates. New research focuses on fabricating these structures on magnetic cores for easier removal, thereby bridging the gap to their industrial application [112,113,114,115,116,117,118,119].

#### 4.1.5. MOF–Polymer Composites

MOF–polymer sorbents are typically designed to combine the porosity and transport pathways of an MOF scaffold with redox-active polymer functionalities that both bind Au^3+^ chloro-complexes and reduce them to immobilized Au^0^/Au^+^. In a Ni–pyrazolate framework (BUT-33) loaded with poly(p-phenylenediamine) (PpPD), Au^3+^ as AuCl_4_^−^ was removed extremely rapidly (>90% in 15 s and >99% in 45 s at 10 ppm) across a pH range of 3–11, with a Langmuir *q*_max_ of about 1600 mg/g and strong selectivity (distribution coefficient for Au about 67,000 mL/g versus approximately 870 mL/g for Cu), consistent with amine-driven redox-chemisorption that forms 3–8 nm Au nanoparticles inside the pores [120].

A sulfur-rich alternative uses poly(thioctic acid) (pTA) confined in Fe-BTC (Fe(III)-benzene-1,3,5-tricarboxylate) pores (Fe-BTC/pTA), achieving >99% Au removal in 10 min near pH 3 (and >95% from pH 3–11) with an experimental capacity around 920 mg/g and a Langmuir fit around 1068 mg/g at 25 °C while maintaining negligible co-ion uptake even in mixed solutions containing many base metal ions at 10-fold higher concentration than Au [121].

Taken together, these studies show that polymer chemistry largely sets the capture mechanism and kinetics. Amine-rich polymers can deliver “seconds-scale” uptake, while sulfur-rich polymers deliver robust selectivity under competitive ions.

To translate fast-batch performance into practical recovery, several works have embedded MOF–polymer into fabrics, granules, or beads that can operate in flow-through reactors.

A Zr-MOF with amino-thiophene linkers immobilized on polydopamine-modified cotton (MOR-3@pda@cotton) combines batch, cartridge flow-through, and passive sampling. It reported *q*_max_ of about 883.5 mg/g for AuCl_4_^−^ and about 43.4 mg/g for PVP-stabilized Au nanoparticles. More than 97% of Au removal in the e-waste model solution was reported in columns, while Cu/Ni/Al uptake was below 7.3% [122].

For continuous processing at higher concentrations, Fe-BTC/PpPD composites structured into 250–500 mm granules with a fluoropolymer binder maintained Au selectivity in tap and creek water [123]. A similar deployment format was developed for Fe-BTC/PpPD-60%-APS in alginate beads. The MOF was effective in extracting gold from CPU pin leachates, showing the best performance at pH 3. Column operation on diluted aqua regia CPU leachate (about 25 ppm Au) produced approximately 604 mg/g at breakthrough and up to around 922 mg/g at about 60% overall recovery, with high selectivity [124].

Across fabric, granule, and bead implementations, the common result is that structuring mainly addresses hydraulics and deployment (flow stability, handling, and concentration capture). Meanwhile, the in-pore polymer redox chemistry still controls Au selectivity. The trade-off is that field-ready formats must be tested for long-term durability (pressure drop, binder or bead stability, and regeneration compatibility) under realistic flow rates and cycle counts.

Overall, MOF–polymer composites consistently leverage redox-chemisorption (polymer sites bind AuCl_4_^−^ and reduce Au^3+^ to immobilized Au^0^/Au^+^), achieving high selectivity in mixed-ion solutions. Engineered deployment formats demonstrate feasible pathways for real-world, continuous gold recovery.

#### 4.1.6. Polymer Sorbents

A freestanding, hierarchically porous poly(imine dioxime) membrane (PIDO) combines sorption with filtration, achieving a Langmuir *q*_max_ of approximately 9250 mg/g (experimental capacity around 8920 mg/g at pH 3) and quantitative Au removal from 1 to 50 mg/L Au solutions (and over 99% from CPU aqua regia leachate). It maintains separation factors between 10,000 and 100,000 for Cu/Ni/Fe and tolerates 0.01–1.0 M mineral acids with minimal performance loss [125].

Beyond single-metal capture, several polymers use pH control and complexation tuning to separate gold from other precious metals in strongly acidic chloride media. A crosslinked polyamidoxime hydrogel (PAO) was specifically configured as a “proton-driven” multistage system for mixed Au/Pd feeds in 0.5–2.0 M HCl. It reported Langmuir capacities of about 2165.75 mg per g for Au^3+^ and 1835.73 mg/g for Pd^2+^ in highly acidic conditions ([H^+^] = 1 mol/L) and a separation factor k(Au)/k(Pd) around 36.5, with approximately 95.4% gold recovery after five cycles via staged binding and elution rather than deliberate in-sorbent redox [126].

Macroporous acrylic acid cryogels (PAA cryogels) specifically target selective uptake within mixed noble metal chloride solutions (Au, Pd, Pt in 0.1–1 M HCl). They exhibit a Au uptake of about 134.7 mg/g at an initial Au concentration of 800 mg/L and demonstrate separation factors of approximately 386 for Au over Ru, and 77 and 133 for Au over Pd and Pt, respectively, including operation in cryogel-packed column formats [127].

These studies collectively show that polymer ion exchange and chelation can be adjusted by acidity and speciation to selectively extract precious metals. However, the most effective systems balance separation performance with metal uptake. For example, cryogels prioritize selectivity and flow form-factor, while PAO focuses on staged Au/Pd separation at very low pH. The trade-off is that multistage systems and column formats increase process complexity, which must be justified by improved downstream purity or easier recovery.

A more common approach is to incorporate solvent-extraction capabilities into solid polymers through warm impregnation, which involves combining the sorbent and extractant at elevated temperatures [128]. A tri-butyl phosphate (TBP)-impregnated polymer resin (Amberlite XAD-16–TBP) extracted AuCl_4_^−^ most effectively at 6 M HCl, with capacities around 145.937–147.64 mg/g (depending on TBP–resin ratio) and Au^3+^ removal of about 97.46% (1:2) or 92.11% (1:3) under the reported low solid-to-liquid tests. Desorption of Au was achieved by stripping with thiourea/HCl, yielding 89.58% or 82.99% efficiency [129].

Similarly, polyamide 6 nanofibers loaded with Cyanex 272, an organophosphinic extractant, achieved over 77% Au extraction from a 20 mg/L model solution at pH 1.5 and a solid-to-liquid ratio (S/L) of 1:700 within 15 min. Subsequent stripping with 1 M HCl and 1 M thiourea recovered the gold. In real PCB aqua regia leachate (27.6 mg/L Au), the process resulted in approximately sixfold enrichment to 173.1 mg/L [130].

Poly(m-phenylenediamine) nanoparticles (PmPD NPs) and their NP-assembled membranes were reported to achieve over 99% Au removal from acidic mixed-metal wastewater (about 1 M HCl) with minimal co-adsorption of Cu/Ni/Zn, reporting a *q*_max_ of about 2063 mg/g for the nanoparticles and a dynamic membrane capacity of approximately 530 mg/g (PmPD) [131].

A photo-assisted variant is polydopamine-coated mesoporous polymer microspheres (PDA-pMS), in which catechol groups chelate AuCl_4_^−^ and reduce Au^3+^ while illumination boosts electron transfer. Under simulated sunlight, more than 99% of 1 mM Au^3+^ was removed within 3 h, and Langmuir capacities increased from about 0.660 mmol/g in the dark (around 130 mg/g) to 2.84 mmol/g under >450 nm light (approximately 560 mg/g) and 6.87 mmol/g under full spectrum (about 1350 mg/g). The process showed high selectivity at a 1 mM concentration of metal chloride ions (Au, Pt, Cu, Co, Ni, Zn, Fe, Al) [132].

Redox-chemisorption polymers consistently demonstrate that reducing Au^3+^ during sorption can prevent competing ions from disrupting gold adsorption.

High selectivity was reported for a low-capacity additively manufactured polyamide 12 adsorbent (AM-N12) in highly acidic chloride media (5 wt.% HCl), with a Langmuir qmax of approximately 5.25 mg/g and about 97% gold recovery in multi-metal tests while most base metals remained in solution [133].

Polymer-based sorbents showed the highest process maturity among different sorbents, having been studied as membranes, columns, or in staged elution processes. Near-quantitative Au removal was observed for PIDO membranes with low co-adsorption of Cu/Ni/Fe ions. Proton-driven polyamidoxime hydrogel system achieved a remarkable 95.4% gold recovery after five staged binding and elution cycles in 0.5–2 M HCl. Amberlite XAD-16-TBP sorbent achieved Au extraction efficiencies of 92–97%, demonstrating the potential of the warm impregnation method for fabricating polymer-based sorbents. On the other hand, Cyanex 272-loaded polyamide 6 nanofibers achieved about 77% gold removal from real PCB aqua regia leachates, followed by thiourea stripping. An extraction efficiency of over 99% was observed for PmPD membranes in 1 M HCl mixed-metal wastewater, with similar results seen for the photo-assisted PDA-pMS sorbent. Finally, low-capacity polymers like AM-N12 removed more than 97% of Au from mixed-metal solutions under highly acidic conditions [125,126,127,128,129,130,131,132,133].

**Table 3:** Examples of sorbent materials for selective gold extraction from chloride solutions.

Sorbent	Feed/Matrix and Key Conditions	Performance	Selectivity/ Competition	Mechanism and Regeneration
COF–PGBpy/PGBD benzoxazine COFs; heteroatom-rich N, O sites [109]	PCB in aqua regia leachate + model AuCl_4_^−^ pH ~0–1; 10–30 min	*q*_max_ 3467 mg/g (PGBpy), 2590 mg/g (PGBD); >92% Au recovery from PCB leachate	Cu, Fe, Ni, Zn, Al present; Au uptake barely affected by 100 times excess co-ions	Chelation + redox on benzoxazine/N sites; 10 and 5 adsorption–desorption cycles reported
COF–Tp-BTD AA/AB/ABC benzothiadiazole COFs; light-responsive [108]	AuCl_4_^−^ 50–100 ppm; pH 2–9; batch 2 mg/10 mL; ≤6 h	*q*_max_ ~2526–3095 mg/g under 460 nm; dark capacities much lower; PSO + Langmuir fits	Simulated smartphone e-waste (Au 6.23 ppm, Cu/Ni hundreds ppm), 97.8% Au, little Cu/Ni	Photo-redox chemisorption; COF electrons reduce Au^3+^ to Au^0^
COP–TFACOP imine COP + COP-180 porphyrin/phenazine polymer [111]	AuCl_4_^−^ solutions + CPU/aqua regia leachates; pH 2–12 (optimum 4–6); batch 10 mg/10 mL; 24–48 h; 25–45 °C	*q*_max_ 2975 mg/g (N-TFACOP, 45 °C); 1620 mg/g (COP-180); >97–99.9% Au removal	Mixed metal feeds with Cu, Co, Ni, Pb; negligible base metal uptake; effective Au/Pt capture	Reductive adsorption on N-rich conjugated frameworks; Au^3+^ to Au^0^; regeneration with thiourea/HCl or HNO_3_/HCl
MOF-Thiolated Zr-MOFs: UiO-66-AT/MSA/TA [114,115,116], UiO-66-SH composites, Zr-MSA-AA [117], PCN-222-MBA [119]	AuCl_4_^−^ in acidic chloride (incl. 3 M HCl); model and e-waste leachates; 10–1000 mg/L; ≤1–120 min	Au^3+^ *q*_max_ ~374–1587 mg/g; up to 1021 mg/g (Zr-MSA-AA) and 714 mg/g (PCN-222); Langmuir + PSO behavior	Highly Au-selective vs. Cu, Ni, Zn, Fe, Pb; high distribution coefficients; tolerates strongly acidic, multi-metal matrices.	Au–S chemisorption with partial reduction of Au^3+^ to Au^0^; regenerable over several cycles using acid/oxidant eluents
Fe-BTC/BUT-33 MOF–polymer composites (PpPD, pTA; powders, granules, beads) [121,123,124]	[AuCl_4_^−^] 1–5000 ppm; tap/creek water, natural waters, CPU/PCB leachates; pH ~3–11; batch + columns; 10–240 min; T ~Room temperature	*q*_max_ ~920–1600 mg/g (Fe-BTC/pTA, BUT-33–PpPD); powder Fe-BTC/PpPD ~934 mg/g; >99% Au removal in dilute feeds	Strong Au preference: *K*_d_(Au) ~6.7 × 10^4^ mL/g vs. ~103 for Cu; Au/Ni ~972; negligible Ca/Mg/Al/Co uptake	Redox chemisorption: PpPD/pTA amine/disulfide sites reduce Au^3+^ to Au^0^ within MOF
Polymer–PIDO membrane; hierarchical porous polymer [125]	AuCl_4_^−^(pH 1–5) and CPU aqua regia leachate; pH adjusted to ~3; membrane filtration; [Au]^0^ ~40 mg/L	Langmuir *q*_max_ 9250 mg/g; experimental ~8920 mg/g; ~100% Au removal from 1–50 mg/L solutions and CPU leachate	Competing Cu, Ni, Fe, Zn, etc., hardly adsorb across wide pH/acid range; strong Au-over-base metal selectivity	Soft-donor chelation + redox at imine- dioxime N, O sites; metallic Au formed; regeneration/eluent not reported
Polymers–PmPD nanoparticles/ membranes + PAO hydrogel (amide/amine-rich) [131]	PmPD: AuCl_4_^−^ in ~1 M HCl e-waste wastewater; PAO: 0.5–2 M HCl leachates with Au^3+^/Pd^2+^; batch/column	*q*_max_ ~2063 mg/g (PmPD NPs); dynamic membrane ~530 mg/g; PAO Langmuir *q*_max_ 2165.75 mg/g (Au^3+^), 1835.73 mg/g (Pd^2+^)	PmPD: minimal Cu/Ni/Zn co-adsorption; PAO: high Au/Pd separation factors; 95.4% Au recovery after 5 cycles	PmPD: electrostatic binding and redox to Au^0^; PAO: proton-responsive amidoxime chelation/ion exchange; multiple adsorption–desorption cycles
CB–Graphene-based carbons: GO/CS sponge, rGO membranes, TU-rGO, rGO@cellulose paper [95,96,97]	AuCl_4_^−^ model solutions and PCB/gold-plating leachates; mildly acidic (pH 1–4); 25 °C; contact time 1–168 h; [Au^0^]: 2–8170 ppm	GO/CS: Au^3+^ 16.8 g/g and Au^+^ 6.2 g/g; rGO membranes: 1797 mg/g; TU-rGO: 833 mg/g; rGO@cellulose: up to 4660 mg/g	Strong Au selectivity vs. Ag, Cu, Co, Ni, Fe, Na, Mg; Au uptake much higher than competing cations	Cooperative chemisorption (GO/CS), thiourea S/Nchemisorption (TU-rGO), cation–π and reductive deposition on rGO; TU-rGO recyclable 5 cycles

#### 4.1.7. Non-Sorbent Recovery Methods

Direct reduction methods are a classic example of selective gold recovery from chloride solutions. Oxalic acid was used to reduce gold ions from the HCl-NaClO_3_ solution, achieving a high reduction efficiency (95.56%) [134]. Additionally, ascorbic acid with K_2_CO_3_ was reported to synthesize the gold particles from pure gold solutions [135].

A novel electrodeposition redox-replacement process provides an advantage over traditional cyanide-based methods by reducing Au from a chloride solution through Cu species deposited on the electrodes under an applied potential. In mixed-metal chloride solutions, Korolev et al. reported a 94.4% Au recovery with a Au product purity of 93.7%, demonstrating strong selectivity in multi-metal solutions [136]. Since chloride solutions are highly acidic and corrosive, selecting the right electrode material is crucial. It was found that stainless steel electrodes offered better performance for Au deposition compared to nickel or titanium, with a Au recovery of 28.1 wt.% under the tested conditions [137].

Another approach involves using ionic liquids, which are organic salts made entirely of ions with melting points below 100 °C. Pyrrolidinium-based ionic liquids have been successfully applied to extract Au^3+^ from real CPU leachates. A representative ionic liquid [Pyr-EA][NTf_2_] (1-(2-ethylamino)pyridinium bis(trifluoromethylsulfonyl)imide) showed high extraction capacity of 524.6 mg/g with satisfactory selectivity toward gold over base metals. More than 5 adsorption/desorption cycles were conducted with minimal decrease in extraction efficiency (84.3% retained capacity after 5 cycles) when using oxalic acid as a desorbing reagent [138]. Dual-function long-chained carboxylate-functionalized imidazolium bromide [C_12_C_1_COOHim]Br showed a synergistic effect of adsorption of gold from aqua regia via anion exchange and as a leachate for copper via coordination on carboxylic acid groups. A gold recovery rate of 96.7% was reported [139].

Although most research on Au removal from chloride systems is shifting toward different sorbent technologies, other hydrometallurgical methods provide comparable extraction efficiencies. For example, the electrodeposition redox-replacement process achieved 94.4% Au recovery with 93.7% metallic gold purity from multi-metal chloride solutions. Similarly, oxalic acid was used to demonstrate 95.56% Au reduction from HCl–NaClO_3_ leachate. Novel ionic-liquid systems also showcase strong Au removal with pyrrolidinium ionic liquids (ILs), demonstrating high extraction capacity and maintained performance (84.3% retained capacity) after five adsorption/desorption cycles. On the other hand, dual-function imidazolium IL was effective at 96.7% Au recovery from aqua regia-derived solutions [134,136,137,138,139].

### 4.2. Alkaline Thiosulfate Leachates (Au-Thiosulfate Complexes)

Gold recovery from ammoniacal thiosulfate solutions is limited by the coprecipitation of copper through cementation or low purity when electrowon. Therefore, sorbent materials such as thiocarbamoyl derivatives of polyallylamine are increasingly studied for their ability to selectively adsorb gold from solution in the presence of anionic copper complexes. In the work by Privar et al., thiocarbamoyl-polyallylamine sorbent achieved 95% gold recovery from model ammonical thiosulfate solutions (40 mg/L precious metal concentrations) [140]. The gold recovery after elution reached 86%, and this performance was sustained over three consecutive adsorption–elution cycles without sorbent regeneration. Another sorbent material that was effective at selectively leaching gold was an anion exchange fiber. Neag et al. studied the effects of different parameters on gold sorption to maximize total recovery [141]. The most influential parameter was the amount of fiber, while the initial gold concentration was also important. The reported Au removal ranged from 31.8% to 88%, depending on the fiber dosage and the initial Au concentration. Elution of gold from such a sorbent was identified as a future research objective.

Ion exchange is an effective method for extracting gold from thiosulfate-rich solutions. An Amberlite IRA-400, a strong-base anion exchange resin with quaternary ammonium groups, showed good adsorption performance at higher ammonia levels and pH values. Characterization showed gold adsorbed as a [Au(S_2_O_3_)_2_]^3–^ anionic complex by exchanging with the Cl^–^ counter ion in the resin’s functional group. Under the representative batch conditions reported by Dong et al., the Au concentration decreased from 20 mg/L to 0.56 mg/L after 120 min at a resin dosage of 3 g/L, resulting in 97.2% Au removal [142]. The gold desorption mechanism was studied by Dong et al., who presented a synergistic role of Na_2_SO_3_ and NaCl solution based on the SO_3_^2–^ reaction with [Au(S_2_O_3_)_2_]^3–^ to generate [Au(S_2_O_3_)(SO_3_)]^3–^, which had low affinity for binding with the resin, thus desorption was achieved [143]. Notably, when NaCl or Na_2_SO_3_ were used alone, Au desorption percentages were reported to be close to zero, while the mixed Na_2_SO_3_ + NaCl system enabled effective desorption across operating conditions.

Similarly to gold extraction from chloride solutions, ionic liquids provide an emerging, sustainable method for recovering gold from thiosulfate solutions. Mahandra et al. introduced a recyclable ionic liquid (Cyphos IL 101/102 diluted in toluene) that achieved 98.4% (IL 101) and 96.4% (IL 102) gold extraction [144]. Gold stripping with NaCl reached 97% (IL 101) and 92% (IL 102). On the other hand, functionalized dicationic ionic liquids offered another approach for gold recovery from highly concentrated thiosulfate solutions. The reported extraction efficiencies exceeded 95% between 25 and 45 °C, with high selectivity for competing ions. Additionally, a 94.4% back-extraction efficiency was achieved with minimal loss of extraction capacity [145].

Overall, ammoniacal thiosulfate systems combined with ion exchange resins can achieve high Au removal and extraction rates, with reported efficiencies typically in the 95–99% range under optimized conditions. Thiocarbamoyl–polyallylamine sorbents achieved 95% Au removal from model ammoniacal thiosulfate solutions, with 86% Au recovery after elution, and retained their performance over three adsorption–elution cycles. Anion-exchange fibers also effectively removed Au from thiosulfate leachates, with extraction efficiencies ranging from 31.8% to 88%, depending on fiber dosage and initial Au levels. Ion exchange resins, Amberlite IRA-400, achieved 97.2% Au removal, with effective desorption using a Na_2_SO_3_ + NaCl eluent. Ionic-liquid extraction offers high performance simultaneously, with Cyphos IL 101/102 reported to achieve 96.4–98.4% Au extraction and 92–97% Au recovery after NaCl stripping. Dicationic ionic liquids achieved over 95% extraction and 94.4% recovery with minimal capacity loss while being reusable [140,141,142,143,144,145].

### 4.3. Iodine-Iodide Leachates

Activated carbon proves highly effective for gold recovery from iodine–iodide leachates where Au exists as in the form of iodide complexes. Leaching waste phone PCBs in iodine-iodide solution resulted in approximately 98% batch adsorption on activated carbon at pH 7, 25 °C, and an initial S/L ratio of 5 g/L. This process achieved 60% Au removal in 15 min, with a total of 2 h. The adsorption mechanism involves pore-filling and surface complexation at oxygen-containing sites [146].

Meng et al. reported an electrodeposition method with a high gold deposition efficiency (94.02%) from iodine leaching solution [147]. The gold was deposited onto the cathode plate through granular gain accumulation, suggesting a potential industrial application.

Activated carbon allows quick and extensive Au removal from iodine–iodide leachates, reaching 98% Au adsorption from waste phone PCB iodide leachate under optimal conditions (pH 7, 25 °C, S/L = 5 g/L), with 60% Au removed within 15 min and nearly complete uptake within 2 h. Similarly, electrodeposition from an iodine-leaching solution achieved a 94.02% gold deposition efficiency, showing an effective downstream recovery method for iodide-based systems [146,147].

**Figure 5:**
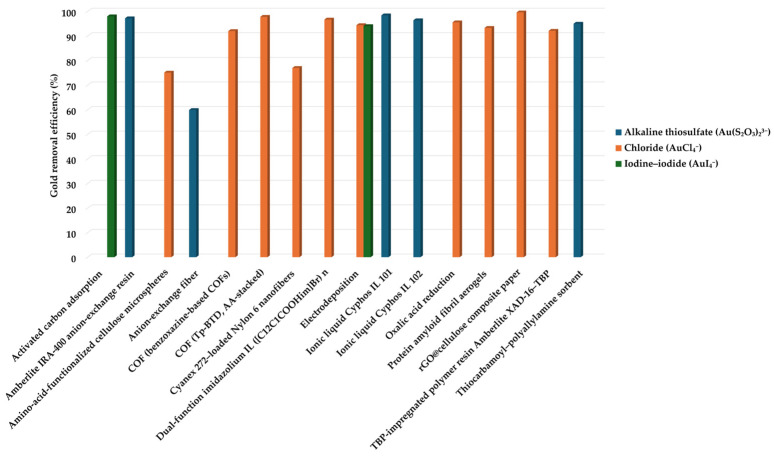
Gold removal efficiency using different recovery systems across leaching families.

Across the reviewed studies (Figure 5), gold recovery from iodine–iodide leachates appears consistently high despite the limited number of extraction processes represented. In contrast, gold extraction systems from thiosulfate leachates show greater spread, indicating that recovery is more strongly conditioned by the selected extraction system than it is uniform across different methods. For extraction from chloride leachates, most systems achieve high performance, but the figure shows that reported efficiencies are method dependent. Electrodeposition extraction delivers among the strongest performance and is comparable to results from iodine-based systems.

## 5. Discussion

**Figure 6:**
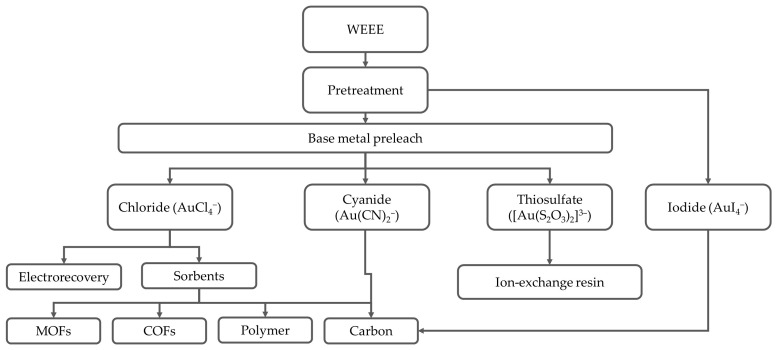
Integrated WEEE hydrometallurgical flowsheet with staged base metal removal, leaching, and gold recovery.

When recovering gold from WEEE, a comprehensive approach is essential, as no single method or material addresses all challenges. Therefore, an integrated process that combines pretreatment, leaching, and recovery in a complementary way is required. The best process maximizes gold yield and purity while reducing energy use, chemical consumption, and waste. An example of an integrated WEEE hydrometallurgical flowsheet, which incorporates staged base metal removal followed by precious-metal recovery, is provided in Figure 6.

Achieving selectivity for gold often comes at the cost of complexity, requiring additional steps to remove base metals or to design a specialized sorbent for subsequent recovery.

For example, removing <90% Cu, the main interfering metal, with nitric acid or sulfuric acid and hydrogen peroxide leaves a residue where non-cyanide lixiviants like thiosulfate and thiourea can then dissolve gold [65,66,67,87,90]. Thus, an integrated flowsheet might intentionally sacrifice the recovery of certain metals to streamline gold extraction.

In a gold recovery process, where WEEE feedstock makes up over 70% of the total intrinsic value, it might be beneficial to eliminate interfering elements early to make subsequent selective steps easier [12].

The example of a 43.8% Au loss when hard disk drives are not dismantled before shredding highlights the risk of improper integration [30]. Modern e-waste recycling facilities increasingly adopt flexible process selection, where high-value streams receive more manual sorting and customized hydrometallurgy, while low-value bulk may be sent to smelting [31]. Flexibility is crucial because WEEE feed composition changes over time (e.g., less gold per phone as electronics become more efficient, more complex alloys, etc.) [1,2,3,4].

Section 4 shows that the best gold recovery method depends on the chemical form of gold in a solution. A key point is that the leaching system and the extraction method must be designed together.

**Figure 7:**
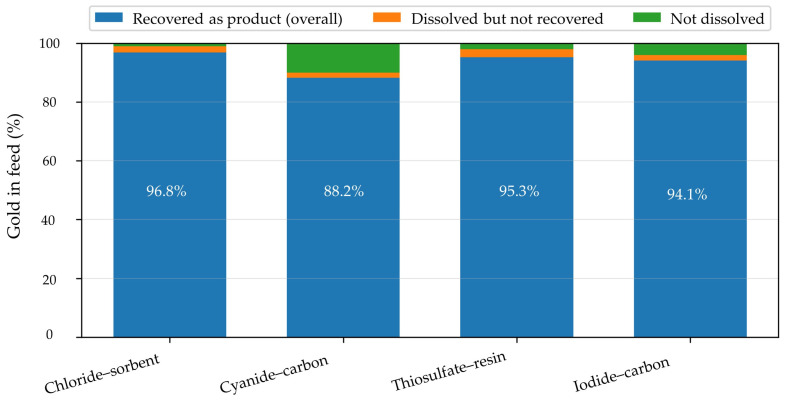
Overall Au recovery from WEEE for representative paired leaching–recovery systems.

For instance, if aqua regia leaching is the preferred method, the downstream recovery process should be applicable in an acidic environment and be selective for competing ions, since aqua regia dissolves all metallic parts [59,60,61,62,63]. For ammoniacal thiosulfate pregnant leach solution, ion exchange extraction is preferred over precipitation due to the risk of coprecipitation of other metals, such as copper [140,141,142,143]. On the other hand, iodide leaching should be combined with activated carbon or electrodeposition methods [146,147]. Thus, each recovery pair has its advantages and disadvantages, and gold recovery efficiency (Figure 7):

- Chloride-sorbent systems allow fast leaching and potentially high gold recoveries but have issues with high corrosivity, toxic emissions, limited selectivity, and the requirement for acid waste treatment [59,60,61,62,63].
- Cyanide-carbon systems remain well established and highly selective for Au(CN)_2_^−^ with straightforward stripping [148] yet depend on highly toxic reagents and often show decreased gold extraction efficiency in copper-rich feeds, requiring pre-leaching steps [23,79,80].
- Thiosulfate–resin systems offer a non-toxic alternative and can achieve selective gold recovery when copper is controlled [85,87,142,143]. However, they are constrained by complex solution chemistry, significant thiosulfate consumption, specialized resin elution requirements, and sensitivity to leaching conditions such as pH and Cu^2+^ concentration [83,84,85,86,87,140,141,142,143].
- Iodide-carbon systems provide high selectivity under relatively mild conditions and allow iodine reuse. Still, their broader implementation is limited by iodine cost and volatility, the need for efficient iodine recovery, limited large-scale validation, and potential safety concerns associated with concentrated I_2_ [77,78,146].

An interesting observation is that most selective leaching processes for gold over base metals, such as copper, often show slower kinetics and incomplete gold dissolution. For example, iodide leaching yielded only 79% of Au in one case but was highly selective for Au relative to base metals. However, implementation may be limited because not all the gold from WEEE is dissolved [78]. In contrast, aqua regia achieves nearly complete dissolution of all metals, both base and precious. The challenge is in selectively recovering gold from the pregnant solution [59,60,61].

When designing a gold recovery system, decisions should be made about whether to prioritize complete recovery at the expense of sustainability. In the context of WEEE recycling, and considering the high value of gold, higher gold yields are sought. However, safety concerns during operation and waste handling when using aqua regia may influence the decision to adopt greener processes at the expense of recovery.

The selection of which process flow to implement depends heavily on the context. For instance, a small recycling plant might opt for a safer, simpler gold recovery method, even if it results in some gold loss. Conversely, larger, better-equipped refiners may aim for maximum recovery because they have the capacity to handle toxic process chemicals.

Many novel sorbent materials (MOFs, COFs, nanocomposites, biosorbents) demonstrate exceptional performance in a single use. However, during continuous operation, their stability and reusability become limiting factors for their adoption [94,95,96,97,98,99,100,101,102,103,104,105,106,107,108,109,110,111,115,116,117,118]. For example, the Zr-based MOF made from mercaptosuccinic acid had a high capacity but lost half of its over 10 cycles [117]. Magnetic composites improve sorbent-based systems by allowing sorbents to be removed magnetically instead of through filtration [118]. However, magnetic cores tend to dissolve in acidic media and need proper protection, which adds to the complexity of the synthesis [149].

A sustainable WEEE recycling process should reduce energy consumption and greenhouse gas emissions. Traditional pyrometallurgical methods are energy-intensive and can produce toxic fumes, but they are simple to handle with mixed WEEE streams [19]. Conversely, hydrometallurgical processes combined with sorption-based systems are carried out under ambient atmospheric conditions [26,27]. However, sorbent synthesis, drying, and sorbent incineration for gold extraction might increase overall energy consumption.

Furthermore, chemical consumption is another important factor to consider, especially in processes that use large amounts of acids, oxidants, or specialized reagents. Additionally, many processes produce secondary waste, as mentioned earlier. Aqua regia leaching yields acidic metal nitrate/chloride solutions requiring neutralization and disposal [59,60,61,62,63]. While thiosulfate leaching is regarded as a greener alternative to cyanide, it tends to break down when in contact with certain metals [84]. In practice, the essential for sustainable gold recovery is a closed-loop reagent cycle, where byproducts from one process are recovered and regenerated to support operations.

From a sustainability standpoint, processes that eliminate cyanide and prevent uncontrolled thermal reduction of waste are more preferable [150]. This review highlights several promising alternative leaching systems to traditional industrial cyanide leaching. For example, the thiosulfate–resin system provides remarkable gold recovery while avoiding cyanide. [151]. Similarly, iodine/iodide is considered safer than aqua regia and cyanide systems, but is more costly [152,153]. Thiourea reduces the acute toxicity of cyanide and can be used to desorb gold from various sorbents [154,155].

To assess the overall sustainability of various process flows, a life-cycle assessment (LCA) should be performed to quantify environmental impacts across different impact categories [156]. For example, a comparative LCA might reveal that pyrometallurgical recovery of gold from WEEE has high CO_2_ emissions but produces less chemical waste. Conversely, although hydrometallurgical recovery results in lower greenhouse gas emissions, it may raise ecotoxicity concerns due to effluent discharge [157].

This study reviews methods for recovering gold from waste electrical and electronic equipment. Nevertheless, gold is not the only target. Recovery of other metals, such as copper, can generate additional revenue streams. Therefore, synergistic recovery of other metals should be considered when designing a WEEE recycling system. Ideally, a cascading system where one process outputs a flow to the next would be most efficient.

While many studies report gold dissolution and recovery efficiencies, cross-study comparisons of energy consumption, environmental impact, scalability, and cost are often difficult because operating conditions, feed composition, and system boundaries vary and are not consistently reported. Nevertheless, some trends can be observed.

High-temperature pyrometallurgical gold recovery processes are industrially mature and can be applied to heterogeneous WEEE feed. On the other hand, the disadvantage is that they are energy-intensive and require the management of toxic gas emissions, such as furans and dioxins [158]. Aqua regia leaching enables nearly quantitative dissolution of WEEE but is constrained by its corrosivity and by the acidic secondary waste generated, which requires neutralization and disposal [159]. Cyanide–carbon systems are well established in gold refining from ore, but due to toxicity, regulatory constraints, and cyanide consumption in copper-rich feeds, they are constrained by operational costs and deployability [28,160]. Halide systems (I_2_ and Br_2_) can achieve high gold dissolution from WEEE and show improved selectivity (iodine-based systems), but their scalability is limited by high reagent costs and volatility unless closed-loop recycling is implemented [62,161]. Thiosulfate-based routes compared to cyanide offer reduced acute toxicity, yet their adoption is constrained by complex solution chemistry, ligand decomposition, and the need for tailored downstream capture and elution processes [162,163].

Emerging sorbents such as MOFs, COFs, biosorbents, and nanocomposites exhibit excellent one-pass capacity and selectivity, but scale-up is limited by sorbent synthesis costs, long-term stability, and viable performance under continuous-flow in real-leaching conditions [98,159,164,165].

For future work and site-specific decision-making, techno-economic analysis and LCA are recommended to compare different flowsheets, using consistent functional units and system boundaries to evaluate potential gold recovery systems from WEEE.

In conclusion, the comparative analysis connects innovations in the pretreatment process to the design of lixiviants and final extraction methods.

The trend in research is moving towards milder, safer chemicals, such as thiosulfate, for selective gold dissolution away from cyanide use. Also, there is an increase in research on new nanoporous sorbent materials and functionalized polymers and biosorbents for selective gold adsorption from real WEEE pregnant leachates.

## 6. Conclusions

This review emphasizes that selective gold recovery from WEEE begins not at the leaching stage but with proper mechanical pretreatment and physical separation. Mechanical recycling processes, including dismantling, comminution, and gravity-, magnetic-, or electrostatic-based separation, are crucial for concentrating gold-bearing fractions, reducing the feed volume, and decreasing interference from base metals before chemical processing. Therefore, the success of subsequent leaching and recovery depends heavily on the quality of the mechanically prepared feed. Subsequently, this review meticulously analyzes a speciation-based approach for the extraction of gold from waste electrical and electronic equipment. It highlights emerging techniques focused on selective dissolution and gold adsorption:

- Efficient gold recovery begins with effective disassembly, comminution, and separation steps that concentrate gold and remove interfering materials. Manual removal of high-value parts and sequential physical separations can increase the gold grade of the leach feed several times, while preventing gold losses to dust and fines. Going forward, pretreatment flowsheets should be chosen based on feed type. For instance, high-grade server motherboards might justify extensive dismantling, whereas low-grade mixed scrap might go straight to bulk mechanical processing. Innovations such as automated sorting (AI-guided vision to pick out gold-rich components) and improved liberation techniques (e.g., cryogenic milling, selective crushing) are expected to further enhance pretreatment efficiency.
- Selective leaching could replace cyanide and high-temperature pyrometallurgy: Several viable alternative lixiviants have been demonstrated for WEEE. Acidic halide systems (HCl + oxidant, bromine, iodine) under optimized conditions match the performance of aqua regia for gold dissolution. Greener alternatives to cyanide, such as thiosulfate and thiourea, show comparable gold yields when proper base metal removal is performed in prior steps.
- Novel sorbents such as covalent organic frameworks, metal–organic frameworks, biosorbents, polymers, and composites offer highly selective gold adsorption from mixed metal solutions. Their application is constrained by limited research on performance under continuous-flow conditions, long-term durability, and cost.
- A potential new WEEE recycling system would combine multiple technologies to recover several metals from waste electronics. A likely scenario involves a modular process: the first stage produces a Cu-rich solution and an Au-enriched solid. In the second stage, gold is leached from the residue using a selective lixiviant, and in the third stage, gold is recovered as a high-purity form product. Remaining residues, such as plastics, glass, or minor metals, are either processed or safely disposed of.

In conclusion, research into gold recovery is advancing toward more sustainable, selective, and modular methods for extracting gold from WEEE. By combining advanced pretreatment with speciation-focused leaching and recovery, it becomes more practical to extract gold with high yield and purity without the environmental impact of traditional methods. The next steps include scaling these solutions, analyzing their economics within real-world recycling systems, and ensuring that improvements in one part of the process do not cause issues in the next. Continued innovation and collaboration among materials scientists, chemical engineers, and recycling practitioners are expected to enable the transition of “green” gold extraction from e-waste from laboratory demonstrations to industrial application. Such progress would significantly advance circular economy goals and reduce reliance on primary gold mining. The approaches reviewed in this study, spanning novel sorbents and alternative lixiviants, establish a foundation for this sustainable transition.

## Figures and Tables

**Table 1:** Common pretreatment methods for e-waste before gold extraction.

Pretreatment Method	Typical Operations	Impact on Au Distribution	Key Features
Disassembly and industrial pre-processing [30,31]	Manual or semi-automated disassembly of WEEE, selective removal of high-value components, industrial lines combining manual disassembly, comminution, screening, and sorting	Au concentration increases by 7 times compared to shredding of whole electronic equipment; separation of high-gold-bearing components (PCBs, ICs, connector pins), mass reduction for subsequent processes	High selectivity at component level, lower downstream chemical use
Mechanical comminution and size classification [7,17,32]	Crushing, milling, and grinding of WEEE; screening into size fractions	Metals liberated from polymer/ceramic, Au concentration increases in intermediate (0.075–0.18 mm) and fine (<0.075 mm) fractions, feed is prepared for physical separation and leaching	Increase in electro-chemical reclamation process capacity, increased energy consumption (fine particles), simple and high throughput, essential for liberation
Gravity-based separation of milled WPCBs [33,34,35,36,37]	Shaking tables, hydrocyclones, dense/heavy media separation, centrifugal gravity separation	Concentration of dense metal-rich fractions, removal of low-density fractions (polymer, glass), reduces mass for downstream leaching	Increase in Au grade with minimal losses, novel sustainable medium (sodium silicate aqueous solution) instead of chloroform for density medium separation: Knelson centrifuges demonstrate high recovery for Au from fines
Magnetic and electrostatic separation [38,39,40]	Wet/dry magnetic separation; electrostatic and inertial separators integrated into dry mechanical flowsheets	Magnetic separation of ferromagnetic metals, electrostatic separation of polymer/ceramics from metals	Not selective for Au (both methods), wet magnetic may separate metallic dust; surfactant (octyl phenol ethoxylate)
Froth flotation/natural floatability [41,42,43]	Reverse flotation or natural floatability separation of PCB powders, sometimes combined with gravity concentration	Metal-rich particles partition from polymer based on surface hydrophobicity, increase in metallic concentrate (Au enrichment), increase in Au content with prior pyrolysis vs. mechanical pretreatment step	Can upgrade fine metallic fractions that are difficult to capture by gravity, reagent-lean process
Thermal pretreatment (pyrolysis, chlorination, supergravity) [44,45,46]	Low-temperature or microwave pyrolysis of WPCBs, pyrolysis plus solid-state chlorination, pyrometallurgical treatment with supergravity separation	Removal of organics and modification of solder/metal phases, Au retained in solid residue	Reduced organic load, need for fine comminution, and volume for hydrometallurgy; energy recovery from gas and oil
Chemical swelling/delamination of waste printed circuit boards (WPCBs) [47]	Organic solvents with microwave-assisted pyrolysis for epoxy/glass delamination	Rapid delamination with minimal mechanical damage improves access to Au in hydrometallurgy; cleaner separation between metallic and non-metallic fractions	Requires solvent-handling infrastructure; potentially toxic and flammable

## Data Availability

No new data were created or analyzed in this study. Data sharing is not applicable to this article.

## Abbreviations

The following abbreviations are used in this manuscript:

ACIL900	Imidazolium ionic-liquid-grafted biomass carbon
AM-N12	Additively manufactured polyamide 12 (nylon 12)
APS	Ammonium persulfate
AR	Aqua regia
BTD	Benzothiadiazole
BUT-33	Nickel–pyrazolate metal–organic framework (Beijing University of Technology)
CB	Carbon-based
COF	Covalent organic framework
COP	Covalent organic polymer
CPU	Central processing unit
CS	Chitosan
DMC	Dithiocarbonate-modified cellulose
e-waste	Electronic waste
Fe-BTC	Fe(III)-benzene-1,3,5-tricarboxylate
GDP	Gross Domestic Product
GO	Graphene oxide
GPU	Graphics processing unit
IC	Integrated circuit
IL	Ionic liquid
JNM	Jinan University MOF series
LCA	Life cycle assessment
MOF	Metal–organic framework
MSA	Mercaptosuccinic acid
NBS	N-bromosuccinimide
PAO	Polyamidoxime
PCB	Printed circuit board
PCN-222	Porphyrinic Zr-MOF
PDA	Polydopamine
PGBD	Phloroglucinol-benzidine COF
PGBpy	Phloroglucinol-4,4′-bipyridine COF
PIDO	Poly(imine-dioxime)
PmPD	Poly(m-phenylenediamine)
PpPD	Poly(p-phenylenediamine)
PSO	Pseudo-second-order
PVP	Poly(vinylpyrrolidone)
RAM	Random access memory
rGO	Reduced graphene oxide
S/L	Solid-to-liquid ratio
TBP	Tri-butyl phosphate
TFACOP	Fluorinated imine-based covalent organic polymer
Tp	1,3,5-triformylphloroglucinol
TpTGCl	Triformylphloroglucinol–triaminoguanidinium chloride
TU	Thiourea
UiO-66	University of Oslo series Zr-based MOF
WEEE	Waste electrical and electronic equipment
Zr-MOF	Zirconium-based MOF
